# Integration of MULTIMOORA algorithm combined with circular q-rung orthopair fuzzy information for optimizing player positioning

**DOI:** 10.1038/s41598-025-18795-0

**Published:** 2025-10-16

**Authors:** Asma Farhad, Kifayat Ullah, Zeeshan Ali, Dragan Pamucar

**Affiliations:** 1 Department of Mathematics, Riphah International University (Lahore Campus), Lahore, 54000 Pakistan; 2 Department of Mathematics, Saveetha School of Engineering, Saveetha Institute of Medical and Technical Sciences, Saveetha University, Chennai, Tamil Nadu 602105 India; 3 Department of Information Management, National Yunlin University of Science and Technology, Yunlin, Douliu, Taiwan, R. O. C.; 4 Department of Operations Research and Statistics, Faculty of Organizational Sciences, University of Belgrade, Belgrade, Serbia; 5 Széchenyi István University, Győr, Hungary

**Keywords:** Circular q-rung orthopair fuzzy information, Dombi t-norm, and t-conorm, Decision analysis process with MULTIMOORA method, Optimizing player positioning, Engineering, Mathematics and computing

## Abstract

The following paper presents a new analytical framework for the optimization of player positioning, a methodology with significant practical implications. The method implements the multi-objective optimization by ratio analysis with full multiplicative form (MULTIMOORA) in a decision-making context in which several non-commensurable performance variables have to be combined. The application of Dombi operationalizes the framework by prioritizing weighted aggregation operators coupled with circular q-rung orthopair fuzzy sets (Cq-ROFSs). The Cq-ROFSs allow multidimensional representation of uncertainty, and allow dynamic actions upon the fuzzy parameter q, such that both intuitionistic fuzzy sets and Pythagorean fuzzy sets are subsets. Two Dombi prioritized operators on Cq-ROFSs are thereby devised a Cq-ROFSs Dombi prioritized weighted averaging operator (Cq-ROFSDPWA) and a Cq-ROFSs Dombi prioritized weighted geometric operator (Cq-ROFSDPWG). Results from empirical experiments are reported that demonstrate the performance of the resulting methodology, highlighting its practical relevance. The fundamental properties of these operators are also examined. The proposed aggregation operators are applied within the MULTIMOORA technique to assess their effectiveness. Numerical examples demonstrate that the methods yield logical and consistent results across different decision-making scenarios. Comparative analyses further highlight the advantages of the Cq-ROFSDPWA and Cq-ROFSDPWG operators over existing approaches.

**Supplementary Information:**

The online version contains supplementary material available at 10.1038/s41598-025-18795-0.

## Introduction

One crucial component of decision sciences is Multi attribute decision-making (MADM), a procedure that can produce ranking outcomes for finite options based on the attribute values of many alternatives^1^. C. Shit and G. Ghorai proposed Dombi aggregation operators under Fermatean fuzzy information for MADM. Shit et al., developed a harmonic aggregation operator for trapezoidal picture fuzzy MADM problems^2^. C. Shit and G. Ghorai applied Aczel-Alsina aggregation with hesitant fuzzy sets to select the best educational brand^3^. Nowadays, MADM is widely used in numerous sectors because it is related to the development of organizations and social decision-making in all its dimensions^4^. Trying to better, effectively, and correctly convey the attribute value is a key issue in real-world decision-making processes^5^. The expression of attribute values of alternatives by exact values is insufficient in the real world due to the vagueness of decision-making contexts and an array of decision-making challenges as well. Zadeh^6^ created the FS theory to address these kinds of problems. FS is made up of the truth grade phrase and the recommendation to resume at the unit interval. However, there are several circumstances where the idea of FS is ineffective. For instance, the FS theory cannot deal with information presented to a person in the form of truth and falsity grades. The way Atanassov^7^ attempted to overcome the limitations of the classical fuzzy sets was to combine the non-membership degree (MD) in a proposition and the measure of membership degree (MD) therein by formulating the intuitionistic Fuzzy Set (IFS) framework. Due to its limitation, whereby the total power of these two parameters lies in the unit interval, IFS assumes a versatile and effective tool in the process of making decisions about complex and suspicious data. The IFS model has thus been used in a wide range of fields by many researchers^7^. C. Shit and G. Ghorai used interval-valued picture fuzzy VIKOR to select charging methods for public stations^8^.

Despite its benefits, the IFS model also exhibits problems whereby the provided values of truth and falsehood sum to more than one. To solve this constraint, Yager^7^ proposed an amendment to the IFS rule, stating that the squared addition of the truth and falsehood coefficients should have its square sum within the [0, 1] interval. This led to the development of the Pythagorean Fuzzy Set (PyFS). Comparative analysis of evidence that PyFS is superior to IFS when it comes to dealing with complex and uncertain data in the decision-making context. Various researchers in many fields, therefore, have embraced the PyFS concept^9^. Based on PyFS, Yager^10^ later studied the circumstances under which PyFS can be interpreted as a q-rung orthopair Fuzzy Set (q-ROFS), that the q-power of any mental form of truth and falsehood is no greater than one. The thus-named q-ROFS framework has drawn a lot of interest because of the flexibility that the q-parameter offers to it, allowing it to be used to diagnose deficiencies in many different applications^11–13^.

More recently, the circular IFS mode came about, which limits its drawing to the domain below the intuitionistic fuzzy interpretation triangle^14^. To increase the representational scope of such a construct, a new extension has been proposed, that of the circular q-rung orthopair fuzzy sets (Cq-ROFS), extending the uncertainty domain to outside the intuitionistic fuzzy interpretation triangle, though being circumscribed therein by the circle. There are many connections and operations, especially mathematical operations, for Cq-ROFS^15–17^.

Karande and Chakraborty^18^ investigated using the conventional Multi-objective optimization based on the ratio analysis (MOORA) approach. Baležentis et al.^10^ employed the MULTIMOORA approach and IFSs for performance management in another investigation. The study’s suggested methodology has been expanded to include other aggregation strategies. Multiple decision-making issue techniques have been extended to IFSs. Aggregation operators are a crucial component in numerous attribute issues (AO). Xu^19^ presented fundamental aggregation operators for IFSs. Based on IFSs, Xu and Yager^20^ created fundamental geometric operators. Using t-norm and t-conorms, formulated aggregation operators such as the Hamacher approach based on interval-valued IFSs^21^. Hussain and Pamucar^22^ presented novel aggregation operators using rough sets. A series of Schweizer-Sklar mathematical approaches developed by Hussain et al.^23^. Jaleel^24^ investigated some reliable agricultural robotics under the system of bipolar fuzzy theory. Advanced decision analysis and database management systems were established in^25^. Hussain et al.^26^ evaluated the performance of Dombi’s mathematical approaches using interval-valued spherical fuzzy theory. Mahmood et al.^27^ put forward the theory of an innovative approach to the spherical fuzzy context. Hussain et al.^28^ deliberated on some new approaches to the Aczel Alsina operations. The advanced technology of the electric motor car was developed by Hussain et al.^29^. Alcantud et al.^30^ discussed a novel approach to temporal intuitionistic fuzzy theory. Hwang et al.^31^ investigated new similarity measures using Sugeno integral operators. Farid et al.^32^ A new technique for waste management was discussed, utilizing the q-rung orthopair fuzzy context. Alreshidi et al.^33^ combined two theories of similarity and entropy measures for deriving new approaches. Bui et al.^34^ proposed a decision-making problem using the properties of similarity measures. However, Archimedean and Einstein operators can also be applied to solve issues involving numerous attributes^35,36^.

Often, decision support systems require considering a wide range of criteria that affect the selection of available options^37,16^. Furthermore, it is difficult to describe how to select the best solutions given the expert’s ambiguity about how to express the linkages between the data that have been gathered. These issues also occur in sports when players or coaches have a wide range of options to choose from while planning a team for a tournament or conducting training. This work suggests an objective fuzzy inference system based on fuzzy logic to evaluate players in team sports using football as an example. For offensive positions, a multi-criteria model based on the characteristics of the MULTIMOORA method has been developed to evaluate players based on their match facts. This method works well for assigning player skill ratings, as the study has shown. The unique qualities of the MULTIMOORA technology led to its selection.

This study has taken a novel method in light of the previously mentioned thorough investigations and literature review. Prioritized aggregation is associated with the Dombi operator. A more comprehensive process based on the q-ROFVs decision matrix is introduced. Moreover, the benefits and capabilities of employing this approach can be summed up in the following ways:

1. Decision-makers are aware that in actual MADM issues, each characteristic has a distinct priority level. Thus, in the present investigation, priority AO is used to indicate various priority levels. Experts or different techniques can be used to calculate the weight values, and an individual’s desired order of priority can be used to identify the priority order. As a result, the criteria’s weighting and priority order are not required to coincide.
2. The decision makers’ evaluations appear as an additional wide range by Cq-ROFVs.
3. A variety of Dombi aggregation operators (AOs) are formulated using the theory of Cq-ROF information and prioritized aggregation operators.
4. The MULTIMOORA method is an effective optimization technique for the MADM method under the system of Cq-ROF context.
5. A MADM approach is explored using newly introduced operators, which are supported by certain numerical examples.
6. The comparison analysis and the impact of the parameters are also examined to assess the reliability and knowledge of the operators under investigation.

The structure of this framework is illustrated in the following ways: In Sect. 2, we examine the concept of Cq-ROFSs and their operational norms. There is also an extensive examination of the Dombi operators. In Sect. 3, we employ the Dombi prioritized weighted aggregation operators and describe their characteristics. In Sect. 4, the specified operators are used with the MULTIMOORA technique. In Sect. 5, the specified operators and their uses are demonstrated using a numerical example. Additionally, comparisons of the suggested models were made for various parameters. The suggested techniques were also contrasted with those of previous research. The final section discusses Future research and the suggested framework’s benefits.

## Preliminaries

To understand basic concepts and fundamental roles, we provide an overview of some notions of q-ROFS and Dombi operations.

### Q-rung orthopair fuzzy set (q-ROFs)

The theory of q-ROFS was developed by Yager^10^ in 2016. We also explored the notion of PFSs^38^ and Fermatean fuzzy sets (FFSs)^39^.

#### **Definition 1**^10^

 A mathematical shape of q-ROFS $\:C$ is given by:

1 $$\:\begin{array}{c}C=\left\{<\mathcalligra {x},\:{\mu\:}_{C}\left(\mathcalligra {x}\right),{\upsilon\:}_{C}\left(\mathcalligra {x}\right)>|\mathcalligra {x}\in\:X\right\}\:\:\end{array}\:\:\:$$

Where $\:{\mu\:}_{C}\left(\mathcalligra {x}\right):X\to\:\left[\text{0,1}\right]$ and $\:{\upsilon\:}_{C}\left(\mathcalligra {x}\right):X\to\:\left[\text{0,1}\right]$ indicate the MD and NMD, respectively, subject to a condition:$\:\:0\le\:{\mu\:}_{C}{\left(\mathcalligra {x}\right)}^{q}+{\upsilon\:}_{C}{\left(\mathcalligra {x}\right)}^{q}\le\:1.$ Moreover, the hesitance value is denoted by$\:\:{\pi\:}_{C}\left(\mathcalligra {x}\right)={\left(1-{\mu\:}_{C}{\left(\mathcalligra {x}\right)}^{q}+{\upsilon\:}_{C}{\left(\mathcalligra {x}\right)}^{q}\right)}^{\frac{1}{q}}$ and the pair$\:\:c=\left(\mu\:,\upsilon\:\right)$ is known as a q-rung orthopair fuzzy value.

#### **Definition 2**^10^

 Consider three q-ROFVs $c=\left(\mu\:,\upsilon\:\right),\:{c}_{1}=\left({\mu\:}_{1},{\upsilon\:}_{1,}\right)$ and $\:{c}_{2}=\left({\mu\:}_{2},{\upsilon\:}_{2}\right)$. Then, basic operations are given as follows:

- $$\:{c}_{1}\oplus{c}_{2}=\left(\sqrt[q]{{\left({\mu\:}_{1}\right)}^{q}+{\left({\mu\:}_{2}\right)}^{q}-{\left({\mu\:}_{1}\right)}^{q}{\left({\mu\:}_{2}\right)}^{q}}\:,\:{\upsilon\:}_{1}{\upsilon\:}_{2}\right)$$
- $$\:{c}_{1}\otimes{c}_{2}=\left(\sqrt[q]{{\left({\mu\:}_{1}\right)}^{q}+{\left({\mu\:}_{2}\right)}^{q}-{\left({\mu\:}_{1}\right)}^{q}{\left({\mu\:}_{2}\right)}^{q}-{\left({\upsilon\:}_{1}\right)}^{q}{\left({\upsilon\:}_{2}\right)}^{q}}\:\:\right)$$
- $$\:{c}_{1}\ominus{c}_{2}=\left(\sqrt[q]{\frac{{\left({\mu\:}_{1}\right)}^{q}-{\left({\mu\:}_{2}\right)}^{q}}{1-{\left({\mu\:}_{2}\right)}^{q}}},\frac{\:{\upsilon\:}_{1}}{{\upsilon\:}_{2}}\right),\:$$if $$\:0\le\:\:\frac{{\upsilon\:}_{1}}{{\upsilon\:}_{2}}\le\:\sqrt[q]{\frac{{\left({\mu\:}_{1}\right)}^{q}-{\left({\mu\:}_{2}\right)}^{q}}{1-{\left({\mu\:}_{2}\right)}^{q}}}\le\:1,$$ otherwise $$\:0$$;
- $$\:\eta.c=\left(\sqrt[q]{1-{\left(-{\mu\:}^{q}\right)}^{\eta}},\:{\upsilon\:}^{\eta}\right)$$, for $$\:\eta>0;$$.
- $$\:{c}^{\eta}=\left(\sqrt[q]{1-{\left(-{\mu\:}^{q}\right)}^{\eta}},\:{\upsilon\:}^{\eta}\right)$$, for $$\:\eta>0;$$.

#### **Definition 3**^40^

Consider a q-ROFV$\:\:c=\left(\mu\:,\upsilon\:\right),\:q\ge\:1$. Then, some expressions of score values and accuracy values are given as follows:

2 $$\:\begin{array}{c}S\left(c\right)=\frac{1}{2}\left(1+{\mu\:}_{c}^{q}-{\upsilon\:}_{c}^{q}\right)\:S\left(c\right)\in\:\left[\text{0,1}\right]\:\end{array}$$

3 $$\:\begin{array}{c}H\left(c\right)={\mu\:}_{c}^{q}+{\upsilon\:}_{c}^{q}\:H\left(c\right)\in\:\left[\text{0,1}\right]\end{array}$$

Here, we discussed a comparison of two different q-ROFVs $\:{c}_{1}$and $\:{c}_{2}$ as follows:

If $\:S\left({c}_{1}\right)<S\left({c}_{2}\right)$ then $\:{(c}_{1})<\left({c}_{2}\right)$; if $\:S\left({c}_{1}\right)=S\left({c}_{2}\right),$ then $\:\left(1\right)$ $\:H\left({c}_{1}\right)=H\left({c}_{2}\right)$ then $\:{c}_{1}={c}_{2}$; $\:\left(2\right)$ if $\:H\left({c}_{1}\right)=H\left({c}_{2}\right)$, then $\:{c}_{1}<{c}_{2}$.

### Dombi operations

This subsection presents some dominant expressions of Dombi t-norm and t-conorm.

#### **Definition 4**^41^

 Consider two positive real numbers $\:\mathcalligra {x}$ and $\:\mathcalligra {y}$. Then, the Dombi TN and TCN are characterized as follows:

4 $$\:\begin{array}{c}TN\left(\mathcalligra {x},\mathcalligra {y}\right)=\frac{1}{1+{\left\{{\left(\frac{1-\mathcalligra {x}}{\mathcalligra {x}}\right)}^{\mathcal {K}}+{\left(\frac{1-\mathcalligra {y}}{\mathcalligra {y}}\right)}^{\mathcal {K}}\right\}}^{\frac{1}{\mathcal {K}}}}\:\end{array}$$

5 $$\:\begin{array}{c}TCN\left(\mathcalligra {x},\mathcalligra {y}\right)=1-\frac{1}{1+{\left\{{\left(\frac{\mathcalligra {x}}{1-\mathcalligra {x}}\right)}^{\mathcal {K}}+{\left(\frac{\mathcalligra {y}}{1-\mathcalligra {y}}\right)}^{\mathcal {K}}\right\}}^{\frac{1}{\mathcal {K}}}}\:\end{array}$$

We have discussed the flexible operations of Dombi TN and TCN operations in the context of q-ROFVs.

#### **Definition 5**^42^

 Consider two q-ROFVs$\:\:{c}_{1}=\left({\mu\:}_{1},{\upsilon\:}_{1}\right)$ and$\:\:{c}_{2}=\left({\mu\:}_{2},{\upsilon\:}_{2}\right)$ with $\:q\ge\:1,\eta>0$. Then, we have:

$$\:{c}_{1}\oplus{c}_{2}=\left(\begin{array}{c}\sqrt[q]{1-\frac{1}{1+{\left\{{\left(\frac{{\mu\:}_{1}^{q}}{1-{\mu\:}_{1}^{q}}\right)}^{\mathcal {K}}+{\left(\frac{{\mu\:}_{2}^{q}}{1-{\mu\:}_{2}^{q}}\right)}^{K}\right\}}^{\frac{1}{K}}}},\\\:\sqrt[q]{\frac{1}{1+{\left\{{\left(\frac{1-{\upsilon\:}_{1}^{q}}{{\upsilon\:}_{1}^{q}}\right)}^{\mathcal {K}}+{\left(\frac{1-{\upsilon\:}_{2}^{q}}{{\upsilon\:}_{2}^{q}}\right)}^{\mathcal {K}}\right\}}^{1/K}}}\end{array}\right)$$

$$\:{c}_{1}\otimes{c}_{2}=\left(\begin{array}{c}\sqrt[q]{\frac{1}{1+{\left\{{\left(\frac{1-{\mu\:}_{1}^{q}}{{\mu\:}_{1}^{q}}\right)}^{\mathcal {K}}+{\left(\frac{1-{\mu\:}_{2}^{q}}{{\mu\:}_{2}^{q}}\right)}^{\mathcal {K}}\right\}}^{\frac{1}{K}}}},\\\:\sqrt[q]{1-\frac{1}{1+{\left\{{\left(\frac{{\upsilon\:}_{1}^{q}}{{1-\upsilon\:}_{1}^{q}}\right)}^{\mathcal {K}}+{\left(\frac{{\upsilon\:}_{2}^{q}}{{1-\upsilon\:}_{2}^{q}}\right)}^{\mathcal {K}}\right\}}^{1/K}}}\end{array}\right)$$

$$\:\eta.\:{c}_{1}=\left(\begin{array}{c}\sqrt[q]{1-\frac{1}{1+{\left\{\eta{\left(\frac{{\mu\:}_{1}^{q}}{{1-\mu\:}_{1}^{q}}\right)}^{\mathcal {K}}\right\}}^{\frac{1}{K}}}},\\\:\sqrt[q]{\frac{1}{1+{\left\{{\eta\left(\frac{1-{\upsilon\:}_{1}^{q}}{{\upsilon\:}_{1}^{q}}\right)}^{\mathcal {K}}\right\}}^{1/K}}}\end{array}\right)$$

$$\:\:{c}_{1}^{\eta}=\left(\begin{array}{c}\sqrt[q]{1-\frac{1}{1+{\left\{\eta{\left(\frac{1-{\mu\:}_{1}^{q}}{{\mu\:}_{1}^{q}}\right)}^{\mathcal {K}}\right\}}^{\frac{1}{K}}}},\\\:\sqrt[q]{\frac{1}{1+{\left\{{\eta\left(\frac{{\upsilon\:}_{1}^{q}}{{1-\upsilon\:}_{1}^{q}}\right)}^{\mathcal {K}}\right\}}^{\frac{1}{K}}}}\end{array}\right)$$

#### **Definition 6**^43^

 Consider a collection of preferences$\:\:H=\{{H}_{1},{H}_{2},\:\dots\:,{H}_{t}\}$ with the linear ordering$\:\:{H}_{1}>{H}_{2}>{H}_{3}>,\dots\:,>{H}_{t}$. So, we denote higher priority with $\:j$ such as $\:{H}_{i}<{H}_{j}.$ However, the power average operators are given by:

6 $$\:\begin{array}{c}PA\left({H}_{\mathcal {K}}\left(\mathcalligra {x}\right)\right)=\sum\:_{u=1}^{t}{\zeta\:}_{u}{H}_{u}\left(\mathcalligra {x}\right)\:\end{array}$$

As,

$$\:{\zeta\:}_{i}=\frac{{{\rm\:X}}_{j}}{\sum\:_{j=1}^{t}{{\rm\:X}}_{j}},\:{{\rm\:X}}_{j}=\prod\:_{i=1}^{j-1}S({P}_{i})\left(j=\text{2,3},\dots\:,t\right),{{\rm\:X}}_{1}=1.$$

#### **Definition 7**^44^

 The mathematical shape of C-IFS $\:{A}_{\mathcal{r}}$ in $\:E$ is given by:

$$A_{\mathcalligra{r}} = \left\{ {\left\langle {\left. {x,\mu _{A} \left( x \right),\upsilon _{A} \left( x \right);\mathcalligra{r}|x \in E} \right\rangle } \right.} \right\}$$

As $\:{\mu\:}_{A}\left(x\right):X\to\:\left[\text{0,1}\right]$ and$\:\:{\upsilon\:}_{A}\left(x\right):X\to\:\left[\text{0,1}\right]$ indicate the MD and NMD with an addition value of the radius of the circle $\:\mathcal{r}\in\:\left[\text{0,1}\right]$ among MD and NMD. Furthermore, the C-IFS must satisfy the condition $\:0\le\:{\mu\:}_{A}\left(x\right)+{\upsilon\:}_{A}\left(x\right)\le\:1$.

Moreover, the hesitancy degree of $\:x$ in $\:{A}_{\mathcal{r}}$ is expressed as:$\:\:{\pi\:}_{A}\left(x\right)=1-{\mu\:}_{A}\left(x\right)-{\nu\:}_{A}\left(x\right)$.

#### **Definition 8**^44^

 The mathematical shape of Cq-ROFS $\:{A}_{\mathcal{r}}$ in $\:E$ is given by:

$$A_{\mathcalligra{r}} = \left\{ {\left\langle {\left. {x,\mu _{A} \left( x \right),\upsilon _{A} \left( x \right);\mathcalligra{r}|x \in E} \right\rangle } \right.} \right\}$$

As $\:{\mu\:}_{A}\left(x\right):X\to\:\left[\text{0,1}\right]$ and$\:\:{\upsilon\:}_{A}\left(x\right):X\to\:\left[\text{0,1}\right]$ indicate the MD and NMD with an addition value of the radius of the circle $\:\mathcal{r}\in\:\left[\text{0,1}\right]$ among MD and NMD. Furthermore, the Cq-ROFSmust satisfy the condition $\:0\le\:{\mu\:}_{A}^{q}\left(x\right)+{\upsilon\:}_{A}^{q}\left(x\right)\le\:1$.

Moreover, the hesitancy degree of $\:x$ in $\:{A}_{\mathcal{r}}$ is expressed as:$\:\:{\pi\:}_{A}\left(x\right)=\sqrt[q]{1-{\mu\:}_{A}^{q}\left(x\right)-{\nu\:}_{A}^{q}\left(x\right)}$. The circular q-rung orthopair fuzzy value (Cq-ROFV) is denoted by $\:c=\left({\mu\:}_{A}\left(x\right),{\upsilon\:}_{A}\left(x\right);\mathcal{r}\left(x\right)\right)$.

## Cq-rung orthopair fuzzy Dombi prioritized aggregation operators

This section formulates some flexible operations of Dombi TN and TCN insight into the Cq-rung orthopair fuzzy context.

### **Definition 9**

Consider$\:\:{c}_{1}=\left(\left({\mu\:}_{1},{\upsilon\:}_{1}\right);{\mathcal{r}}_{1}\right)\:,\:{c}_{2}=\left(\left({\mu\:}_{2},{\upsilon\:}_{2}\right);{\mathcal{r}}_{2}\right)$ are two Cq-ROFVs with$\:\:q\ge\:1,\:\eta>0$. Then, we have the following operations:

$$\:{c}_{1}\oplus{c}_{2}=\left(\begin{array}{c}\left(\begin{array}{c}\sqrt[q]{1-\frac{1}{1+{\left\{{\left(\frac{{\mu\:}_{1}^{q}}{1-{\mu\:}_{1}^{q}}\right)}^{\mathcal {K}}+{\left(\frac{{\mu\:}_{2}^{q}}{1-{\mu\:}_{2}^{q}}\right)}^{\mathcal {K}}\right\}}^{1\backslash\:\mathcal {K}}}},\\\:\sqrt[q]{\frac{1}{1+{\left\{{\left(\frac{1-{\upsilon\:}_{1}^{q}}{{\upsilon\:}_{1}^{q}}\right)}^{\mathcal {K}}+{\left(\frac{1-{\upsilon\:}_{2}^{q}}{{\upsilon\:}_{2}^{q}}\right)}^{\mathcal {K}}\right\}}^{1/\mathcal {K}}}}\end{array}\right);\\\:\sqrt[q]{1-\frac{1}{1+{\left\{{\left(\frac{{\mathcal{r}}_{1}^{q}}{1-{\mathcal{r}}_{1}^{q}}\right)}^{\mathcal {K}}+{\left(\frac{{\mathcal{r}}_{2}^{q}}{1-{\mathcal{r}}_{2}^{q}}\right)}^{\mathcal {K}}\right\}}^{1/K}}}\end{array}\right)$$

$$\:{c}_{1}\otimes{c}_{2}=\left(\begin{array}{c}\left(\begin{array}{c}\sqrt[q]{\frac{1}{1+{\left\{{\left(\frac{1-{\mu\:}_{1}^{q}}{{\mu\:}_{1}^{q}}\right)}^{\mathcal {K}}+{\left(\frac{1-{\mu\:}_{2}^{q}}{{\mu\:}_{2}^{q}}\right)}^{\mathcal {K}}\right\}}^{\frac{1}{K}}}},\\\:\sqrt[q]{1-\frac{1}{1+{\left\{{\left(\frac{{\upsilon\:}_{1}^{q}}{{1-\upsilon\:}_{1}^{q}}\right)}^{\mathcal {K}}+{\left(\frac{{\upsilon\:}_{2}^{q}}{{1-\upsilon\:}_{2}^{q}}\right)}^{\mathcal {K}}\right\}}^{\frac{1}{K}}}}\end{array}\right);\\\:\sqrt[q]{\frac{1}{1+{\left\{{\left(\frac{1-{\mathcal{r}}_{1}^{q}}{{\mathcal{r}}_{1}^{q}}\right)}^{\mathcal {K}}+{\left(\frac{1-{\mathcal{r}}_{2}^{q}}{{\mathcal{r}}_{2}^{q}}\right)}^{\mathcal {K}}\right\}}^{1/K}}}\end{array}\right)$$

$$\:\eta.\:{c}_{1}=\left(\begin{array}{c}\left(\begin{array}{c}\sqrt[q]{1-\frac{1}{1+{\left\{\eta{\left(\frac{{\mu\:}_{1}^{q}}{{1-\mu\:}_{1}^{q}}\right)}^{\mathcal {K}}\right\}}^{\frac{1}{K}}}},\\\:\sqrt[q]{\frac{1}{1+{\left\{{\eta\left(\frac{1-{\upsilon\:}_{1}^{q}}{{\upsilon\:}_{1}^{q}}\right)}^{\mathcal {K}}\right\}}^{\frac{1}{K}}}}\end{array}\right);\\\:\sqrt[q]{1-\frac{1}{1+{\left\{\eta{\left(\frac{{\mathcal{r}}_{1}^{q}}{{1-\mathcal{r}}_{1}^{q}}\right)}^{\mathcal {K}}\right\}}^{1/K}}}\end{array}\right)$$

$$\:\:{c}_{1}^{\eta}=\left(\begin{array}{c}\left(\begin{array}{c}\sqrt[q]{1-\frac{1}{1+{\left\{\eta{\left(\frac{1-{\mu\:}_{1}^{q}}{{\mu\:}_{1}^{q}}\right)}^{\mathcal {K}}\right\}}^{\frac{1}{K}}}},\\\:\sqrt[q]{\frac{1}{1+{\left\{{\eta\left(\frac{{\upsilon\:}_{1}^{q}}{{1-\upsilon\:}_{1}^{q}}\right)}^{\mathcal {K}}\right\}}^{\frac{1}{K}}}}\end{array}\right);\\\:\sqrt[q]{1-\frac{1}{1+{\left\{\eta{\left(\frac{1-{\mathcal{r}}_{1}^{q}}{{\mathcal{r}}_{1}^{q}}\right)}^{\mathcal {K}}\right\}}^{\frac{1}{K}}}}\end{array}\right)$$

### Cq-rung orthopair fuzzy Dombi prioritized weighted averaging aggregation operators

This subsection presents a series of new mathematical approaches of the Cq-ROFDPA and Cq-ROFDPWA operators with some feasible properties.

#### **Definition 10**

Let$\:\:{P}_{j}=\left(\left({\mu\:}_{j},{\upsilon\:}_{j}\right);{\mathcal{r}}_{j}\right)\:(j=\text{1,2},\dots\:,t)$ be the collection of Cq-ROFVs. The Cq-ROFDPA operator is expressed as follows:

$$\:Cq-ROFDPA\left({P}_{1},{P}_{2},\dots\:,\:{P}_{t}\right)={\oplus\:}_{j=1}^{t}\left(\frac{{{\rm\:X}}_{j}}{\sum\:_{j=1}^{t}{{\rm\:X}}_{j}}{P}_{j}\right)=\frac{{{\rm\:X}}_{1}}{\sum\:_{j=1}^{t}{{\rm\:X}}_{1}}{P}_{1}\oplus\frac{{{\rm\:X}}_{2}}{\sum\:_{j=1}^{t}{{\rm\:X}}_{2}}{P}_{2}\oplus\:\dots\:\oplus\frac{{{\rm\:X}}_{t}}{\sum\:_{j=1}^{t}{{\rm\:X}}_{j}}{P}_{t}$$

Where $\:{{\rm\:X}}_{j}=\prod\:_{i=1}^{j-1}S({P}_{i}\left)\right(j=\text{2,3},\dots\:,t)$ and $\:{{\rm\:X}}_{1}=1.$ $\:S\left({P}_{i}\right)$ is the value of the score function.

#### **Theorem 1**

Let a collection of Cq-ROFVs$\:\:{P}_{j}=\left(\left({\mu\:}_{j},{\upsilon\:}_{j}\right);{\mathcal{r}}_{j}\right)\:(j=\text{1,2},\dots\:,t)$. Then fused value by using the Cq-ROFDPA operator is also a Cq-ROFV and we have:

$$\:Cq-ROFDPA\left({P}_{1},{P}_{2},\dots\:,\:{P}_{t}\right)=\frac{{{\rm\:X}}_{1}}{\sum\:_{j=1}^{t}{{\rm\:X}}_{1}}{P}_{1}\oplus\frac{{{\rm\:X}}_{2}}{\sum\:_{j=1}^{t}{{\rm\:X}}_{2}}{P}_{2}\oplus\dots\:\oplus\frac{{{\rm\:X}}_{t}}{\sum\:_{j=1}^{t}{{\rm\:X}}_{j}}{P}_{t}$$

7 $$\:\begin{array}{c}=\left(\begin{array}{c}\left(\begin{array}{c}\sqrt[q]{1-\frac{1}{1+{\left\{\sum\:_{j=1}^{t}\left(\frac{{{\rm\:X}}_{j}}{\sum\:_{j=1}^{t}{{\rm\:X}}_{j}}\right){\left(\frac{{\mu\:}_{j}^{q}}{1-{\mu\:}_{j}^{q}}\right)}^{\mathcal {K}}\right\}}^{\frac{1}{K}}}},\\\:\sqrt[q]{\frac{1}{1+{\left\{\sum\:_{j=1}^{t}\left(\frac{{{\rm\:X}}_{j}}{\sum\:_{j=1}^{t}{{\rm\:X}}_{j}}\right){\left(\frac{1-{\upsilon\:}_{j}^{q}}{{\upsilon\:}_{j}^{q}}\right)}^{\mathcal {K}}\right\}}^{\frac{1}{K}}}}\end{array}\right);\\\:\sqrt[q]{1-\frac{1}{1+{\left\{\sum\:_{j=1}^{t}\left(\frac{{{\rm\:X}}_{j}}{\sum\:_{j=1}^{t}{{\rm\:X}}_{j}}\right){\left(\frac{{\mathcal{r}}_{j}^{q}}{1-{\mathcal{r}}_{j}^{q}}\right)}^{\mathcal {K}}\right\}}^{\frac{1}{\mathcal {K}}}}}\end{array}\right)\:\end{array}$$

Where $\:{{\rm\:X}}_{j}=\prod\:_{i=1}^{j-1}S({P}_{i}\left)\right(j=\text{2,3},\dots\:,t)$ and $\:{{\rm\:X}}_{1}=1.$ $\:S\left({P}_{i}\right)$ is the value of the score function.

*Proof* See Appendix A.

#### **Theorem 2**

Let $\:{P}_{j}=\left(\left({\mu\:}_{j},{\upsilon\:}_{j}\right);{\mathcal{r}}_{j}\right)\:(j=\text{1,2},\dots\:,t)$ be the collection of Cq-ROFVs. Where $\:{{\rm\:X}}_{j}=\prod\:_{i=1}^{j-1}S({P}_{i}\left)\right(j=\text{2,3},\dots\:,t)$ and for $\:j=1$, $\:{{\rm\:X}}_{1}=1.$ $\:S\left({P}_{i}\right)$ is the value of the score function. If $\:{P}_{i}$ are all equal. Then $\:{P}_{i}=P$ for all $\:\:i$. We prove the following statement.

$$\:Cq-ROFDPA\left({P}_{1},{P}_{2},\dots\:,\:{P}_{t}\right)=P$$

*Proof* See Appendix B.

#### **Theorem 3**

Let $\:{P}_{j}=\left(\left({\mu\:}_{j},{\upsilon\:}_{j}\right);{\mathcal{r}}_{j}\right)\:\left(j=\text{1,2},\dots\:,t\right)$ be the collection of Cq-ROFVs. Then,$\:{P}^{-}\le\:Cq-ROFDPA\left({P}_{1},{P}_{2},\dots\:,\:{P}_{t}\right)\le\:{P}^{+}.$

*Proof* See Appendix C.

#### **Theorem 4**

Let $\:{P}_{j}$ and $\:{P}_{j}^{{\prime\:}}\left(j=\text{1,2},\dots\:,t\right)$ be two collections of Cq-ROFVs. If $\:{P}_{j}\le\:{P}_{j}^{{\prime\:}}$ for all $j$, where ${{\rm\:X}}_{j}$ $\:=\prod\:_{i=1}^{j-1}S\left({P}_{i}\right)\left(j=\text{2,3},\dots\:,t\right).$ $\:{{\rm\:X}}_{j}^{{\prime\:}}=\prod\:_{i=1}^{j-1}S\left({P}_{i}^{,}\right)(j=\text{2,3},\dots\:,t)$ and ${{\rm\:X}}_{1}={{\rm\:X}}_{1}^{{\prime\:}}=1.S\left({P}_{i}\right)$ and $S\left({P}_{i}^{{\prime\:}}\right)$ are values score function ${P}_{i}$ and ${P}_{i}^{{\prime\:}}$ respectively. Then, $Cq-ROFDPA\:\left({P}_{1},{P}_{2},\dots\:,\:{P}_{t}\right)\le\:Cq-ROFDPA\:\left({P}_{1}^{,},{P}_{2}^{,},\dots\:,\:{P}_{t}^{,}\right)$.

*Proof* See Appendix D.

#### **Definition 11**

Let $\:{P}_{j}=\left(\left({\mu\:}_{j},{\upsilon\:}_{j}\right);{\mathcal{r}}_{j}\right)\:(j=\text{1,2},\dots\:,t)$ be the collection of Cq-ROFVs. $\:\eta={\left({\eta}_{1},{\eta}_{2},\dots\:,{\eta}_{t}\right)}^{T}$ such that $\:{\eta}_{j}>0.\:\:q\ge\:1$ and $\:\sum\:_{j=1}^{\mathcal{r}}{\eta}_{j}=1$ Cq-ROFDPWA operator defined

$$\:Cq-ROFDPWA\:\left({P}_{1},{P}_{2},\dots\:,\:{P}_{t}\right)={\oplus\:}_{j=1}^{t}\left(\frac{{\eta}_{j}{{\rm\:X}}_{j}}{\sum\:_{j=1}^{t}{{\rm\:X}}_{j}}{P}_{j}\right)$$

$$\:=\frac{\:{\eta}_{1}{{\rm\:X}}_{1}}{\sum\:_{j=1}^{t}{{\rm\:X}}_{1}}{P}_{1}\oplus\frac{\:{\eta}_{2}{{\rm\:X}}_{2}}{\sum\:_{j=1}^{t}{{\rm\:X}}_{2}}{P}_{2}\oplus\:...\oplus\frac{{\:{\eta}_{t}{\rm\:X}}_{t}}{\sum\:_{j=1}^{t}{{\rm\:X}}_{j}}{P}_{t}$$

8 $$\:\begin{array}{c}=\left(\begin{array}{c}\left(\begin{array}{c}\sqrt[q]{1-\frac{1}{1+{\left\{\sum\:_{j=1}^{t}\left(\frac{{\eta}_{j}{{\rm\:X}}_{j}}{\sum\:_{j=1}^{t}{{\rm\:X}}_{j}}\right){\left(\frac{{\mu\:}_{j}^{q}}{1-{\mu\:}_{j}^{q}}\right)}^{\mathcal {K}}\right\}}^{\frac{1}{\mathcal {K}}}}},\\\:\sqrt[q]{\frac{1}{1+{\left\{\sum\:_{j=1}^{t}\left(\frac{{\eta}_{j}{{\rm\:X}}_{j}}{\sum\:_{j=1}^{t}{{\rm\:X}}_{j}}\right){\left(\frac{1-{\upsilon\:}_{j}^{q}}{{\upsilon\:}_{j}^{q}}\right)}^{\mathcal {K}}\right\}}^{\frac{1}{\mathcal {K}}}}}\end{array}\right);\\\:\sqrt[q]{1-\frac{1}{1+{\left\{\sum\:_{j=1}^{t}\left(\frac{{\eta}_{j}{{\rm\:X}}_{j}}{\sum\:_{j=1}^{t}{{\rm\:X}}_{j}}\right){\left(\frac{{\mathcal{r}}_{j}^{q}}{1-{\mathcal{r}}_{j}^{q}}\right)}^{\mathcal {K}}\right\}}^{\frac{1}{\mathcal {K}}}}}\end{array}\right)\:\end{array}$$.

where $\:{{\rm\:X}}_{j}$ $\:=\prod\:_{i=1}^{j-1}S\left({P}_{i}\right)\left(j=\text{2,3},\dots\:,t\right)$ and $\:{{\rm\:X}}_{1}=1.S\left({P}_{i}\right)$ is the value of the score function.

### Cq-rung orthopair fuzzy Dombi prioritized weighted geometric aggregation operators

This section formulated an innovative approach of the Cq-ROFDPG and Cq-ROFDPWG operators under the system of Cq-ROF information.

#### **Definition 12**

Let $\:{P}_{j}=\left(\left({\mu\:}_{j},{\upsilon\:}_{j}\right);{\mathcal{r}}_{j}\right)\:(j=\text{1,2},\dots\:,t)$ be the collection of Cq-ROFVs. $\:\text{C}\text{q}-\text{R}\text{O}\text{F}\text{D}\text{P}\text{G}:{\text{q}}^{t}\to\:q$ operator defined as

$$\:Cq-ROFDPG\left({P}_{1},{P}_{2},\dots\:,\:{P}_{t}\right)={\otimes}_{j=1}^{t}\left({P}_{j}^{\frac{{{\rm\:X}}_{j}}{\sum\:_{j=1}^{t}{{\rm\:X}}_{j}}}\right)={P}_{1}^{\frac{{{\rm\:X}}_{1}}{\sum\:_{j=1}^{t}{{\rm\:X}}_{1}}}\otimes{P}_{2}^{\frac{{{\rm\:X}}_{2}}{\sum\:_{j=1}^{t}{{\rm\:X}}_{2}}}\otimes\:...\otimes{P}_{t}^{\frac{{{\rm\:X}}_{t}}{\sum\:_{j=1}^{t}{{\rm\:X}}_{j}}\:\:\:\:\:}$$

where $\:{{\rm\:X}}_{j}$ $\:=\prod\:_{i=1}^{j-1}S\left({P}_{i}\right)\left(j=\text{2,3},\dots\:,t\right)$ and $\:{{\rm\:X}}_{1}=1.S\left({P}_{i}\right)$ is the value of the score function.

#### **Theorem 5**

Let a collection of Cq-ROFVs$\:\:{P}_{j}=\left(\left({\mu\:}_{j},{\upsilon\:}_{j}\right);{\mathcal{r}}_{j}\right)\:(j=\text{1,2},\dots\:,t)$. Then the fused value by using the Cq-ROFDPG operator is also a Cq-ROFV and we have:

$$\:Cq-ROFDPG\left({P}_{1},{P}_{2},\dots\:,\:{P}_{t}\right)={\otimes}_{j=1}^{t}\left({P}_{j}^{\frac{{{\rm\:X}}_{j}}{\sum\:_{j=1}^{t}{{\rm\:X}}_{j}}}\right)$$

$$\:={P}_{1}^{\frac{{{\rm\:X}}_{1}}{\sum\:_{j=1}^{t}{{\rm\:X}}_{1}}}\otimes{P}_{2}^{\frac{{{\rm\:X}}_{2}}{\sum\:_{j=1}^{t}{{\rm\:X}}_{2}}}\otimes\:...\otimes{P}_{t}^{\frac{{{\rm\:X}}_{t}}{\sum\:_{j=1}^{t}{{\rm\:X}}_{j}}}$$

9 $$\:\begin{array}{c}=\left(\begin{array}{c}\left(\begin{array}{c}\sqrt[q]{\frac{1}{1+{\left\{\sum\:_{j=1}^{t}\left(\frac{{{\rm\:X}}_{j}}{\sum\:_{j=1}^{t}{{\rm\:X}}_{j}}\right){\left(\frac{1-{\mu\:}_{j}^{q}}{{\mu\:}_{j}^{q}}\right)}^{\mathcal {K}}\right\}}^{\frac{1}{\mathcal {K}}}}},\\\:\sqrt[q]{1-\frac{1}{1+{\left\{\sum\:_{j=1}^{t}\left(\frac{{{\rm\:X}}_{j}}{\sum\:_{j=1}^{t}{{\rm\:X}}_{j}}\right){\left(\frac{{\upsilon\:}_{j}^{q}}{{1-\upsilon\:}_{j}^{q}}\right)}^{\mathcal {K}}\right\}}^{\frac{1}{\mathcal {K}}}}}\end{array}\right);\\\:\sqrt[q]{1-\frac{1}{1+{\left\{\sum\:_{j=1}^{t}\left(\frac{{{\rm\:X}}_{j}}{\sum\:_{j=1}^{t}{{\rm\:X}}_{j}}\right){\left(\frac{1-{\mathcal{r}}_{j}^{q}}{{\mathcal{r}}_{j}^{q}}\right)}^{\mathcal {K}}\right\}}^{\frac{1}{\mathcal {K}}}}}\end{array}\right)\:\end{array}$$

where $\:{{\rm\:X}}_{j}$ $\:=\prod\:_{i=1}^{j-1}S\left({P}_{i}\right)\left(j=\text{2,3},\dots\:,t\right)$ and $\:{{\rm\:X}}_{1}=1.S\left({P}_{i}\right)$ is the value of the score function.

*Proof* See Appendix E.

#### **Theorem 6**

Let $\:{P}_{j}=\left(\left({\mu\:}_{j},{\upsilon\:}_{j}\right);{\mathcal{r}}_{j}\right)\:(j=\text{1,2},\dots\:,t)$ be the collection of Cq-ROFVs. Where $\:{{\rm\:X}}_{j}=\prod\:_{i=1}^{j-1}S({P}_{i}\left)\right(j=\text{2,3},\dots\:,t)$ and for $\:j=1$, $\:{{\rm\:X}}_{1}=1.$ $\:S\left({P}_{i}\right)$ is the value of the score function. If $\:{P}_{i}$ are all equal. As $\:{P}_{i}=P$ for all $\:\:i$.

Then $$\:Cq-ROFDPG\left({P}_{1},{P}_{2},\dots\:,\:{P}_{t}\right)=P$$.

*Proof* is similar to the proof of theorem 2.

#### **Theorem 7**

Let $\:{P}_{j}=\left(\left({\mu\:}_{j},{\upsilon\:}_{j}\right);{\mathcal{r}}_{j}\right)\:\left(j=\text{1,2},\dots\:,t\right)$ be the collection of Cq-ROFVs. Then,$\:{P}^{-}\le\:Cq-ROFDPG\:\left({P}_{1},{P}_{2},\dots\:,\:{P}_{t}\right)\le\:{P}^{+}.$

*Proof* is similar to the proof of theorem 2.

#### **Theorem 8**

Let $\:{P}_{j}$ and $\:{P}_{j}^{{\prime\:}\:}\left(j=\text{1,2},\dots\:,t\right)$ be two collections of Cq-ROFVs. If $\:{P}_{j}\le\:{P}_{j}^{{\prime\:}}$, For all $\:j$, where$\:\:\:{{\rm\:X}}_{j}$ $\:=\prod\:_{i=1}^{j-1}S\left({P}_{i}\right)\left(j=\text{2,3},\dots\:,t\right).$ $\:{{\rm\:X}}_{j}^{{\prime\:}}=\prod\:_{i=1}^{j-1}S\left({P}_{i}^{,}\right)(j=\text{2,3},\dots\:,t)$ and $\:{{\rm\:X}}_{1}={{\rm\:X}}_{1}^{{\prime\:}}=1.S\left({P}_{i}\right)$ and $\:S\left({P}_{i}^{{\prime\:}}\right)$ are value score function $\:{P}_{i}$ and $\:{P}_{i}^{{\prime\:}}$ respectively. Then, $\:Cq-ROFDPG\:\left({P}_{1},{P}_{2},\dots\:,\:{P}_{t}\right)\le\:Cq-ROFDPA\:\left({P}_{1}^{,},{P}_{2}^{,},\dots\:,\:{P}_{t}^{,}\right)$.

$$\:Cq-ROFDPWG\left({P}_{1},{P}_{2},\dots\:,\:{P}_{t}\right)={\otimes}_{j=1}^{t}\left({P}_{j}^{\frac{{\eta}_{j}{{\rm\:X}}_{j}}{\sum\:_{j=1}^{t}{{\rm\:X}}_{j}}}\right)$$

$$\:={P}_{1}^{\frac{{\eta}_{1}{{\rm\:X}}_{1}}{\sum\:_{j=1}^{t}{{\rm\:X}}_{1}}}\otimes{P}_{2}^{\frac{{\eta}_{2}{{\rm\:X}}_{2}}{\sum\:_{j=1}^{t}{{\rm\:X}}_{2}}}\otimes\:...\otimes{P}_{t}^{\frac{{\eta}_{t}{{\rm\:X}}_{t}}{\sum\:_{j=1}^{t}{{\rm\:X}}_{j}}}$$

10 $$\:\begin{array}{c}=\left(\begin{array}{c}\left(\begin{array}{c}\sqrt[q]{\frac{1}{1+{\left\{\sum\:_{j=1}^{t}\left(\frac{{\eta}_{j}{{\rm\:X}}_{j}}{\sum\:_{j=1}^{t}{{\rm\:X}}_{j}}\right){\left(\frac{1-{\mu\:}_{j}^{q}}{{\mu\:}_{j}^{q}}\right)}^{\mathcal {K}}\right\}}^{\frac{1}{\mathcal {K}}}}},\\\:\sqrt[q]{1-\frac{1}{1+{\left\{\sum\:_{j=1}^{t}\left(\frac{{\eta}_{j}{{\rm\:X}}_{j}}{\sum\:_{j=1}^{t}{{\rm\:X}}_{j}}\right){\left(\frac{{\upsilon\:}_{j}^{q}}{{1-\upsilon\:}_{j}^{q}}\right)}^{\mathcal {K}}\right\}}^{\frac{1}{\mathcal {K}}}}}\end{array}\right);\\\:\sqrt[q]{\frac{1}{1+{\left\{\sum\:_{j=1}^{t}\left(\frac{{\eta}_{j}{{\rm\:X}}_{j}}{\sum\:_{j=1}^{t}{{\rm\:X}}_{j}}\right){\left(\frac{1-{\mathcal{r}}_{j}^{q}}{{\mathcal{r}}_{j}^{q}}\right)}^{\mathcal {K}}\right\}}^{\frac{1}{\mathcal {K}}}}}\end{array}\right)\end{array}$$

*Proof* is similar to the proof of Theorem 2.

## MADM method based on the investigated operators

This section presents the decision algorithm for the derived mathematical approaches of the Cq-ROFDPWA and Cq-ROFDPWG operators. An advanced technique of the MULTIMORA method is used to evaluate some flexible optimal options by combining the theory of criteria and different alternatives. To serve this purpose, consider a set of alternatives. $\:\left\{{\delta\:}_{1},{\delta\:}_{2},\dots\:,{\delta\:}_{m}\right\}$ and a collection of attributes $\:\left\{{\gamma\:}_{1},{\gamma\:}_{2},\dots\:,{\gamma\:}_{t}\right\}$ with weight vectors $\:{\{\eta}_{1},{\eta}_{2},\dots\:,{\eta}_{n}\}$ that satisfies $\:\sum\:_{j=1}^{t}{\eta}_{j}=1$ and $\:{\eta}_{j}\in\:\left[\text{0,1}\right]$. Furthermore, decision-makers assume Cq-ROF information in different alternatives and attributes, which are listed in the decision matrix$\:\:{R}_{ij}={\left[{\mathcal{r}}_{ij}\right]}_{m\mathcalligra {x}t}$. To aggregate given information about different preferences, we use the following algorithm of the MULTIMOORA method and derive mathematical terminologies under the system of Cq-ROF environment.

**Step 1. Problem formulation**

First, the decision maker arranges various attributes and information associated with each alternative or individual in a decision matrix $\:R={\left[{\mathcal{r}}_{ij}\right]}_{m\mathcalligra {x}t}$.

**Step 2. Normalization of Cq-ROF decision matrix**

Mostly, attribute information has two types: benefit attributes (B) and cost type attributes (C). If there is more than one type of attribute information, then we have to normalize the given information using the following expression:

11 $$\:\begin{array}{c}S={s}_{ij}=\left\{\begin{array}{c}{\mathcal{r}}_{ij},j\in\:B\:\\\:neg\:\left({\mathcal{r}}_{ij}\right),j\in\:\\\:C\end{array}\right\}\:\end{array}$$

**Step 3. MULTIMOORA ratio system calculation**

Investigate the ratio system for the MULTIMOORA method using the derived approaches of the Cq-ROFDPWA operator based on Cq-ROF information:

$$\:{\rho\:}_{i}^{RS}=Cq-ROFDPWA\:\left({S}_{ij}\:|\:\text{1,2},\dots\:,t;\eta\right)$$

12 $$\:\begin{array}{c}=\left(\begin{array}{c}\left(\begin{array}{c}\sqrt[q]{1-\frac{1}{1+{\left\{\sum\:_{j=1}^{t}\left(\frac{{\eta}_{j}{{\rm\:X}}_{ij}}{\sum\:_{j=1}^{t}{{\rm\:X}}_{ij}}\right){\left(\frac{{\mu\:}_{ij}^{q}}{1-{\mu\:}_{ij}^{q}}\right)}^{\mathcal {K}}\right\}}^{\frac{1}{\mathcal {K}}}}},\\\:\sqrt[q]{\frac{1}{1+{\left\{\sum\:_{j=1}^{t}\left(\frac{{\eta}_{j}{{\rm\:X}}_{ij}}{\sum\:_{j=1}^{t}{{\rm\:X}}_{ij}}\right){\left(\frac{1-{\upsilon\:}_{ij}^{q}}{{\upsilon\:}_{ij}^{q}}\right)}^{\mathcal {K}}\right\}}^{\frac{1}{\mathcal {K}}}}}\end{array}\right);\\\:\sqrt[q]{1-\frac{1}{1+{\left\{\sum\:_{j=1}^{t}\left(\frac{{\eta}_{j}{{\rm\:X}}_{ij}}{\sum\:_{j=1}^{t}{{\rm\:X}}_{ij}}\right){\left(\frac{{\mathcal{r}}_{ij}^{q}}{1-{\mathcal{r}}_{ij}^{q}}\right)}^{\mathcal {K}}\right\}}^{\frac{1}{\mathcal {K}}}}}\end{array}\right)\end{array}$$

$$\:=\left(\left({\mu\:}_{i}^{RS},{\upsilon\:}_{I}^{RS}\right);{\mathcal{r}}_{i}^{RS}\right),\:i=\text{1,2},\dots\:,m$$

Utilize the following expression to investigate single-term information associated with each alternative:

13 $$\:\begin{array}{c}{\rho\:}_{i}^{RS\:}=\frac{1+{\left({\mu\:}_{i}^{RS}\right)}^{q}-{\left({\upsilon\:}_{i}^{RS}\right)}^{q}-{\left({\mathcal{r}}_{i}^{RS}\right)}^{q}}{2},\:i=\text{1,2},\dots\:,m\:\end{array}$$

Here, crisp values are normalized using the following expression:

14 $$\:\begin{array}{c}{\stackrel{-}{\rho\:}}_{i}^{RS}=\frac{{\rho\:}_{i}^{RS}}{{\text{m}\text{a}\text{x}}_{i}\:{\rho\:}_{i}^{RS}},\:i=\text{1,2},\dots\:,m\:\end{array}$$

Where$$\:\:{\rho\:}_{i}^{RS}\in\:\left[\text{0,1}\right]$$.

**Step 4. Reference point analysis**

Using the theory of the Chebyshev distance formula, compute different reference points for each alternative or individual as follows:

$$\:R=\left({\mathcal{r}}_{1},{\mathcal{r}}_{2},\dots\:,{\mathcal{r}}_{t}\right)=\left(\left({\mu\:}_{1}^{{\prime\:}},{\upsilon\:}_{1}^{{\prime\:}}\right);{\mathcal{r}}_{1}^{{\prime\:}}\right),\left(\left({\mu\:}_{2}^{{\prime\:}},{\upsilon\:}_{2}^{{\prime\:}}\right);{\mathcal{r}}_{2}^{{\prime\:}}\right),\dots\:,\left(\left({\mu\:}_{t}^{{\prime\:}},{\upsilon\:}_{t}^{{\prime\:}}\right);{\mathcal{r}}_{t}^{{\prime\:}}\right)$$

15 $$\:\begin{array}{c}{\mathcal{r}}_{j}=\left(\left({\mu\:}_{1}^{{\prime\:}},{\upsilon\:}_{i}^{{\prime\:}}\right);{\mathcal{r}}_{i}^{{\prime\:}}\right),\:\\\:{\mu\:}_{j}^{{\prime\:}}={\text{m}\text{a}\text{x}}_{i}{\mu\:}_{ij}\:,\:{\upsilon\:}_{j}^{{\prime\:}}={\text{m}\text{i}\text{n}}_{i}{\upsilon\:}_{ij}\:j=\text{1,2},..,t\:\end{array}$$

If an ideal solution has maximum membership degrees $\:R=\left({\mathcal{r}}_{1},{\mathcal{r}}_{2},\dots\:,{\mathcal{r}}_{t}\right)=\left(\left(\text{1,0},0\right),\left(\text{1,0},0\right),\dots\:,(\text{1,0},0)\right).$ Here, we compute the Hamming distance among two different Cq-ROFVs using the following expression: Let$\:\:{F}_{1}=\left(\left({\mu\:}_{1},{\upsilon\:}_{1},{\pi\:}_{1}\right);{\mathcal{r}}_{1}\right)$ and $\:{F}_{2}=\left(\left({\mu\:}_{2},{\upsilon\:}_{2},{\pi\:}_{2}\right);{\mathcal{r}}_{2}\right)$ be two Cq-ROFVs.

16 $$\:\begin{array}{c}{d}_{Hamming\:}\left(A,B\right)=\\\:\frac{1}{2}\left(\frac{\left|{\mathcal{r}}_{a}-{\mathcal{r}}_{b}\right|}{\sqrt{2}}+\frac{1}{2}\left|{\mu\:}_{1}^{q}-{\mu\:}_{2}^{q}\right|+\left|{\upsilon\:}_{1}^{q}-{\upsilon\:}_{2}^{q}\right|\right)\:\end{array}$$

17 $$\:\begin{array}{c}{d}_{ij}={d}_{Hamming}\left({\eta}_{j}{s}_{ij},{\eta}_{j}{\mathcal{r}}_{j}\right),i=\text{1,2},\dots\:,m,\\\:\:j=\text{1,2},\dots\:,m,\:j=\text{1,2},\dots\:,t.\:\end{array}$$

Additionally, compute the maximum Chebyshev distance using the investigated reference point for each alternative or individual:

$$\:{\rho\:}_{i}^{RP}={\text{m}\text{a}\text{x}}_{j}{d}_{ij}$$

Since computed reference point is a non-compensatory technique. The lower value of the reference point $\:{\stackrel{-}{\rho\:}}_{i}^{RP}$ has higher efficiency. Next, we will find out the normalized score.

18 $$\:\begin{array}{c}{\stackrel{-}{\rho\:}}_{i}^{RP}=\frac{{min}_{i}{\rho\:}_{i}^{RP}}{{\rho\:}_{i}^{RP}},\:i=\text{1,2},\dots\:,m.\:\end{array}$$

Where is the highest utility value $\:{\stackrel{-}{\rho\:}}_{i}^{RP}\in\:\left[\text{0,1}\right]$.

**Step 5. Multiplicative utility function evaluation**

Compute the multiplicative utility function using the derived approaches of the Cq-ROFDPWG operator with the degree of weights$\:\:\eta={\left({\eta}_{1},{\eta}_{2},\dots\:,{\eta}_{t}\right)}^{T},\:{\eta}_{j}>0,q\ge\:1$ criteria.

The following expression computes the utility function:

$$\:{\rho\:}_{i}^{MF\:}=Cq-ROFDPWG\:\left({S}_{ij}\:|\:\text{1,2},\dots\:,t;\eta\right)$$

19 $$\:\begin{array}{c}=\left(\begin{array}{c}\left(\begin{array}{c}\sqrt[q]{\frac{1}{1+{\left\{\sum\:_{j=1}^{t}\left(\frac{{\eta}_{j}{{\rm\:X}}_{ij}}{\sum\:_{j=1}^{t}{{\rm\:X}}_{ij}}\right){\left(\frac{1-{\mu\:}_{ij}^{q}}{{\mu\:}_{ij}^{q}}\right)}^{\mathcal {K}}\right\}}^{\frac{1}{K}}}},\\\:\sqrt[q]{1-\frac{1}{1+{\left\{\sum\:_{j=1}^{t}\left(\frac{{\eta}_{j}{{\rm\:X}}_{j}}{\sum\:_{j=1}^{t}{{\rm\:X}}_{j}}\right){\left(\frac{{\upsilon\:}_{ij}^{q}}{{1-\upsilon\:}_{ij}^{q}}\right)}^{\mathcal {K}}\right\}}^{\frac{1}{K}}}}\end{array}\right);\\\:\sqrt[q]{\frac{1}{1+{\left\{\sum\:_{j=1}^{t}\left(\frac{{\eta}_{j}{{\rm\:X}}_{ij}}{\sum\:_{j=1}^{t}{{\rm\:X}}_{ij}}\right){\left(\frac{1-{\mathcal{r}}_{ij}^{q}}{{\mathcal{r}}_{ij}^{q}}\right)}^{\mathcal {K}}\right\}}^{\frac{1}{\mathcal {K}}}}}\end{array}\right)\:\end{array}$$

20 $$\:\begin{array}{c}=\left(\left({{\upmu\:}}_{\text{i}}^{\text{M}\text{F}},{{\upupsilon\:}}_{\text{i}}^{\text{M}\text{F}}\right);{\mathcal{r}}_{\text{i}}^{\text{M}\text{F}}\right)\:i=\text{1,2},\dots\:,m.\:\end{array}$$

Utilize the score function to compute the single-term results using the following expression:

21 $$\:\begin{array}{c}{\rho\:}_{i}^{MF}=\frac{1}{2}\left(1+{\left({\mu\:}_{i}^{MF}\right)}^{q}-{\left({\upsilon\:}_{i}^{MF}\right)}^{q}-{\left({\mathcal{r}}_{i}^{MF}\right)}^{q}\right)\\\:\:i=\text{1,2},\dots\:,m.\:\end{array}$$

Then crisp values are normalized.

22 $$\:\begin{array}{c}{\stackrel{-}{\rho\:}}_{i}^{MF}=\frac{{\rho\:}_{i}^{MF}}{{ma\mathcalligra {x}}_{i}{\rho\:}_{i}^{MF}},\:i=\text{1,2},\dots\:,m.\:\end{array}$$

The maximum value of the computed results$\:\:{\stackrel{-}{\rho\:}}_{i}^{MF}\in\:\left[\text{0,1}\right]$.

**Step 6. Ranking and final decision**

Finally, to evaluate the rank of the alternative, we apply the Dominance theory seen in the three Moora^45^.

**Fig. 1:**
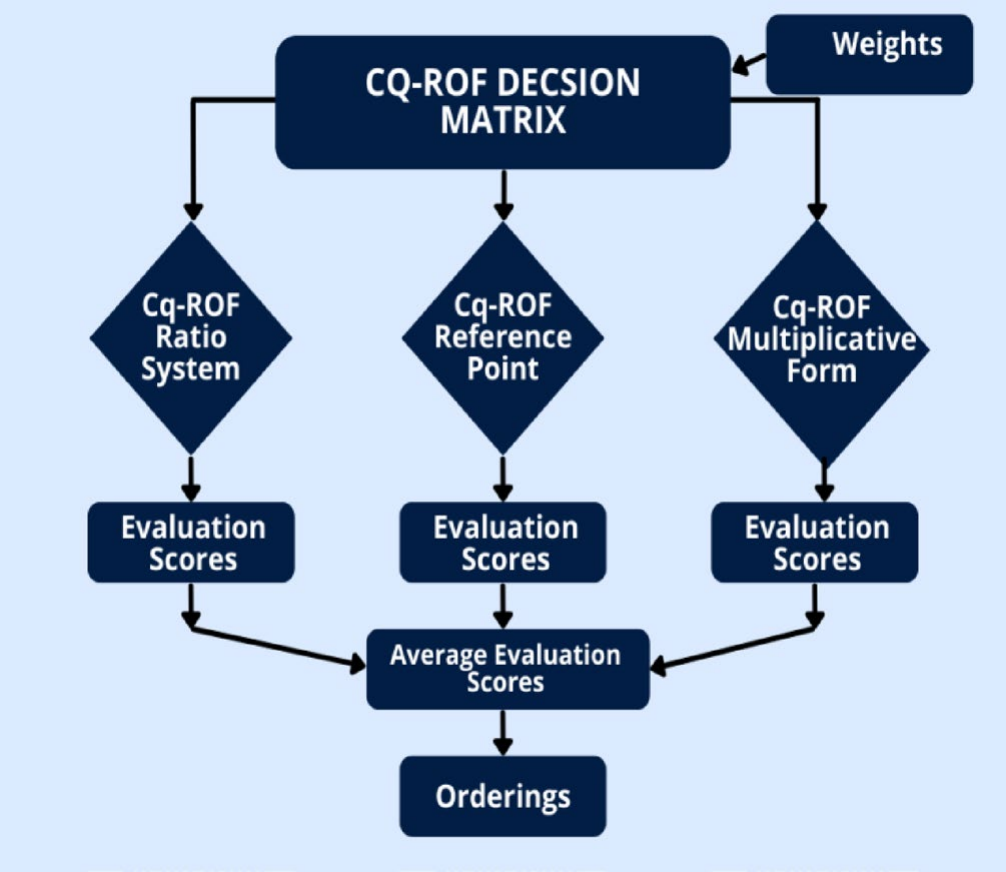
A framework of the MULTIMOORA methodology.

Figure 1 shows the flowchart of the MULTIMOORA method.

## Application

In the context of a football match, one of the most critical factors determining whether a team will emerge victorious is the composition of its players or the positioning of each player within the team that corresponds with the skills he possesses. Generally, the method of selection is performed individually, with the trainer’s estimations serving as the basis for the procedure. In this manner, it is not feasible to determine the value of a structure or team that possesses a particular player foundation. The authors of this research developed a decision support system. The purpose of this system is to provide the coach with guidance in determining which players are the most suitable to fill a particular position within the team structure. The ability of an individual to analyze the ball is a skill that is tested throughout the game of football. There are a lot of individuals all over the world who enjoy playing and watching football. Engaging in the activity of watching or playing football can serve as an effective way of relieving stress brought on by various responsibilities at the workplace, on campus, and beyond. This practice can also be used to strengthen familial connections, which is another potential benefit.

Those who are passionate about football frequently have particular players, organizations, and national teams. Without a doubt, a club’s goal is to win in several different competitions. Many factors contribute to the success of a team, including the lineup of the players, the vision of the head coach, the selection of structures, the distinct capabilities of all players, and other key elements. The club meets all of these responsibilities through the implementation of routine training activities that are meticulously organized, beginning with fundamental drills and involving participation in teamwork. In general, a player is regarded as outstanding if they can play in multiple positions, or more specifically, if they can play in various roles. Because of this limitation, a player can rotate in any circumstance at any time according to the plan that the coach established. Sanata Dharma University (USD FC) is a participant in the football tournaments that the Yogyakarta City PSSI branch organization conducts. In 2015, USD FC was recognized for this distinction. Although the team aims to achieve the First distinction, it faces challenges, including players who are often late and workout routines that conflict with their academic commitments. The growth of the players is impeded as a result of these challenges, and it is difficult for coaches to evaluate and report the players’ performance accurately. There is also a common issue that arises among players who practice regularly and players who practice infrequently. Some players who regularly attend practice sessions are left out when it is time to play for the team in matches, which can make their teammates jealous.

**Fig. 2:**
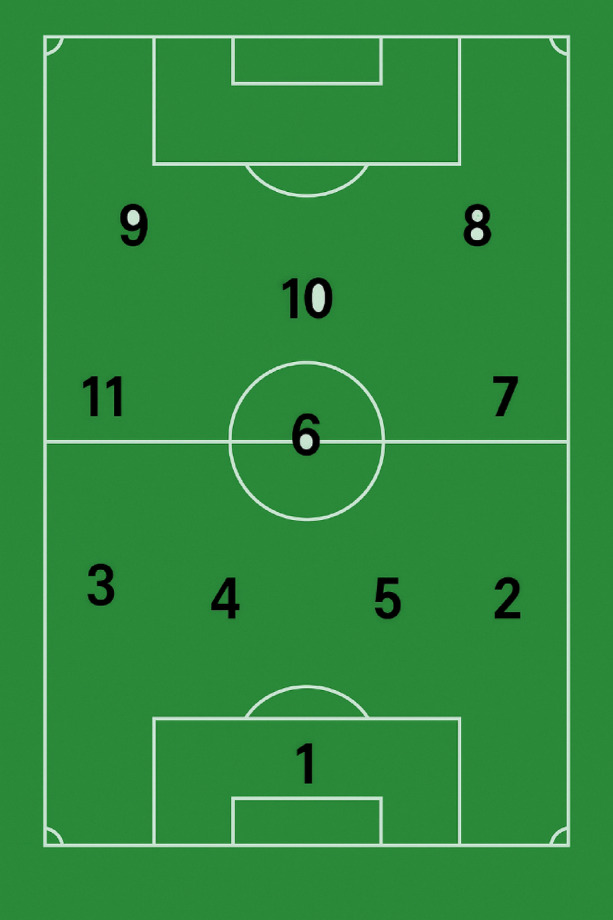
Schematic illustration of the traditional soccer position numbering system (1–11) on a standard field. Numbers represent standard player positions in a classic formation. Figure created by the authors using artificial intelligence.

The numerous barriers mentioned above result in the player’s evaluation data becoming extensive, which can confuse the coach when determining the most suitable player positions and alternative positions based on the capabilities of the players. In the example below, we will examine how the coach can easily assess which players are qualified for specific positions and which players, given their abilities, would be better suited for other positions. Figure 2 illustrates the original positioning numbering Scheme used in football player positioning.

## Numerical example

This section provides real-world examples to illustrate the exactness and continuity of the suggested techniques. Every real-world example is displayed using the MULTIMOORA-Cq-ROF approach. For this example, the impact of the Dombi parameter $\:\mathcal {K}$ and $\:q$ parameters is also examined. We evaluate the findings in light of previous research in the field. The proposed operators were studied in MULTIMOORA-Cq-ROF Pythagorean fuzzy numbers.

### *Example 1*

The purpose of this is to establish the ideal positioning for five football players $\:\left({\mathcal {P}}_{1},{\mathcal {P}}_{2},{\mathcal {P}}_{3},{\mathcal {P}}_{4},{\mathcal {P}}_{5}\right)$or the attacking position at attacking center middle fielder, depending on four essential attributes. These attributes are important for measuring the general efficiency and role played by all players in the squad. We thoroughly examine the abilities and versatility of players for various positions on the playing field. According to the following four attributes are as follows: $\:\left(1\right)\:{E}_{1}$ is the Technical skills; $\:\left(2\right)\:{E}_{2\:}$is the Physical Fitness; $\:{\left(3\right)\:E}_{3}$ is the Tactical Awareness; $\:\left(4\right)\:{E}_{4}$ is Teamwork. Here, we attempt to discover the ideal positions for each player. This procedure is crucial for boosting team effectiveness, increasing player performance, and developing future game planning. By an organized analysis and weighting technique, we give an extensive foundation enabling responsible decision-making in player positioning. A panel of experts, including the head coach, deputy instructors, and the team athletics psychiatrist, was assembled to analyze the players. The panel followed a systematic decision-making process to guarantee certain the assessment was extensive and impartial. The specification of the Prioritization relation attributes is as follows: $\:{E}_{1}>{E}_{2}>{E}_{3}>{E}_{4}>{E}_{5}$. The decision maker assigns some specific degree of weight to the criteria associated to each alternative$\:\:\eta={\left(\text{0.2,0.4,0.5,0.6}\right)}^{T}$. Table 1 shows the decision matrix of the Cq-ROF information. An expert assesses each option in this decision matrix based on the standards in the PFN’s situation. An example is shown for $\:q=3$ and $\:\mathcal {K}=2\:$in the phases that follow. Additionally, the Prioritized matrix for $\:\mathcal {K}=2$ and $\:q=3$ is determined in this way.

$$\:\eta\:PFNs=\left[\begin{array}{c}1\\\:1\\\:1\\\:1\end{array}\:\:\begin{array}{c}0.4865\\\:0.4190\\\:0.4960\\\:0.2625\end{array}\:\:\begin{array}{c}0.3091\\\:0.1642\\\:0.2881\\\:0.0813\end{array}\:\:\begin{array}{c}0.1504\\\:0.0509\\\:0.0636\\\:0.0348\end{array}\right]$$

**Phase (1)** identification of the attributes is of the same type, so there is no need for a normalization process.

**Phase (2)** In this phase, the ratio system for MULTIMOORA-Cq-ROFS is operated on using the Cq-ROFDWA operator. The weight vector of the attributes is $\:\eta={\left(\text{0.2,0.4,0.5,0.6}\right)}^{T}$ Such that $\:{\eta}_{i}>0,q\ge\:1$.

$$\:{\rho\:}_{i}^{RS}=Cq-ROFDPWA\:\left({S}_{ij}\right|\:j=\text{1,2},\dots\:,4;\eta)$$

$$\:=\left(\left({\mu\:}_{i}^{RS},{v}_{i}^{RS}\right);{\mathcal{r}}_{i}^{RS}\right)$$

$$\:=\left[\begin{array}{c}0.6295,\:0.2422,\:0.5664;\:\\\:0.3597,\:0.4000,\:0.5816;\:\\\:0.4623,\:0.2206,\:0.5060;\\\:0.6632,\:0.2197,\:0.6597;\:\\\:0.3833,\:0.3136,\:0.7959\end{array}\right]$$

$$\:i=\text{1,2},\dots\:,5$$

(The outcomes of the aggregation process is $\:\left[\left(\left({\mu\:}_{1}^{RS},{\nu\:}_{1}^{RS}\right);{\mathcal{r}}_{1}^{RS}\right)\right]$, $\left(\left({\mu\:}_{2}^{RS},{\nu\:}_{2}^{RS}\right);{\mathcal{r}}_{2}^{RS}\right),\dots\:,\left(\left({\mu\:}_{5}^{RS},{\nu\:}_{5}^{RS}\right);{\mathcal{r}}_{5}^{RS}\right)$.

**Phase (3)** Investigate the score function using the information of phase 2.

$$\:{\rho\:}_{i}^{RS}=\left(0.0268,-0.1071,-0.0207,\:-0.0030,\:-0.2393\right)$$

The values of the crisp are normalized.

$$\:{\stackrel{-}{\rho\:}}_{i}^{RS}=\frac{{\rho\:}_{i}^{RS}}{{ma\mathcalligra {x}}_{i}{\rho\:}_{i}^{RS}},\:i=\text{1,2},\dots\:,5.$$

$$\:{\stackrel{-}{\rho\:}}_{i}^{RS}=\left(1,-3.9985,-0.7743,\:-0.1128,\:-8.9351\right)$$

For MULTIMOORA-Cq-ROFS the rank result of the ratio system $\:{\mathcal {P}}_{1}>{\mathcal {P}}_{4}>{\mathcal {P}}_{3}>{\mathcal {P}}_{2}>{\mathcal {P}}_{5}$.

For all possibilities, the Chebyshev distance is computed and the reference point is established.

$$\:{\mathcal{r}}_{j}=\left(\left({\mu\:}_{1}^{{\prime\:}},{\upsilon\:}_{i}^{{\prime\:}}\right);{\mathcal{r}}_{i}^{{\prime\:}}\right),\:{\mu\:}_{j}^{{\prime\:}}={\text{m}\text{a}\text{x}}_{i}{\mu\:}_{ij}\:,\:{\upsilon\:}_{j}^{{\prime\:}}={\text{m}\text{i}\text{n}}_{i}{\upsilon\:}_{ij}\:j=\text{1,2},..,4$$

$$\:{d}_{ij}={d}_{Hamming}\left({\eta}_{j}{s}_{ij},{\eta}_{j}{\mathcal{r}}_{j}\right),i=\text{1,2},\dots\:,m,\:j=\text{1,2},\dots\:,5,\:j=\text{1,2},\dots\:,4.$$

Moreover, the maximum Chebyshev distance from the reference point is also calculated for every alternative.

$$\:{\rho\:}_{i}^{RP}={\text{m}\text{a}\text{x}}_{j}{d}_{ij}=\left(0.5268,\:0.3929,\:0.4793,\:0.4970,\:0.2607\right)$$

The process used as a reference point is non-compensatory. Thus, the lesser $\:{\rho\:}_{i}^{RS}$ value has a larger utility. However, normalized utility scores can be computed in the following stage.

**Phase (4)** Here, we computed normalized utility scores using the following expressions:

$$\:{\stackrel{-}{\rho\:}}_{i}^{RP}=\frac{{min}_{i}{\rho\:}_{i}^{RP}}{{\rho\:}_{i}^{RP}}=\left(1,\:0.7459,\:0.9098,\:0.9434,\:0.4949\right)\:$$

$$\:i=\text{1,2},\dots\:,4.$$

Investigate the rank of alternatives using the MULTIMOORA method and Cq-ROF information as follows: $\:{\mathcal {P}}_{1}>{\mathcal {P}}_{4}>{\mathcal {P}}_{3}>{\mathcal {P}}_{2}>{\mathcal {P}}_{5}$

**Phase (5)** In this phase, the multiplicative utility function for MULTIMOORA-Cq-ROFS is treated with the Cq-ROFDPWG operator. Some weight is assigned to each attribute$\:\:\eta={\left(\text{0.2,0.4,0.5,0.6}\right)}^{T}$ such that $\:{\eta}_{i}>0,q\ge\:1$.

$$\:{\rho\:}_{i}^{MF}=Cq-ROFDPWG\:\left({S}_{ij}|\:j=\text{1,2},\dots\:,4;\eta\right)\:\:$$3

$$\:=\left(\left({\mu\:}_{i}^{MF},{v}_{i}^{MF}\right);{\mathcal{r}}_{i}^{MF}\right)$$

$$\:=\left[\begin{array}{c}0.4397,\:0.3529,\:0.4754;\:\\\:0.3139,\:0.3981,\:0.5243;\\\:0.2447,\:0.4439,\:0.4201;\\\:0.1362,\:0.5557,\:0.3652;\:\\\:0.2984,\:0.3763,\:0.4668\end{array}\right]$$

$$\:i=\text{1,2},\dots\:,5$$

$$\:{\rho\:}_{i}^{MF}=\left(0.4668,\:0.4119,\:0.4265,\:0.3911,\:0.4358\right)$$

**Table 1:** Decision matrix with circular pythagorean fuzzy numbers.

$\:\varvec{\mathcal {P}}/\varvec{x}$	$\:{\varvec{E}}_{1}$	$\:{\varvec{E}}_{2}$	$\:{\varvec{E}}_{3}\:$	$\:{\varvec{E}}_{4}$
$\:{\varvec{\mathcal {P}}}_{1}$	$$\:\:\left(\begin{array}{c}\left(\text{0.6,0.3}\right);\\\:0.6\end{array}\right)$$	$$\:\left(\begin{array}{c}\left(\text{0.7,0.2}\right);\\\:0.4\end{array}\right)$$	$$\:\left(\begin{array}{c}\left(\text{0.5,0.3}\right);\\\:0.5\end{array}\right)$$	$$\:\left(\begin{array}{c}\left(\text{0.3,0.5}\right);\\\:0.6\end{array}\right)$$
$\:{\varvec{\mathcal {P}}}_{2}$	$$\:\left(\begin{array}{c}\left(\text{0.3,0.4}\right);\\\:0.5\end{array}\right)$$	$$\:\left(\begin{array}{c}\left(\text{0.4,0.4}\right);\\\:0.6\end{array}\right)$$	$$\:\left(\begin{array}{c}\left(\text{0.3,0.4}\right);\\\:0.7\end{array}\right)$$	$$\:\left(\begin{array}{c}\left(\text{0.5,0.2}\right);\\\:0.5\end{array}\right)$$
$\:{\varvec{\mathcal {P}}}_{3}$	$$\:\left(\begin{array}{c}\left(\text{0.5,0.2}\right);\\\:0.4\end{array}\right)$$	$$\:\left(\begin{array}{c}\left(\text{0.2,0.5}\right);\\\:0.5\end{array}\right)$$	$$\:\left(\begin{array}{c}\left(\text{0.4,0.5}\right);\\\:0.4\end{array}\right)$$	$$\:\left(\begin{array}{c}\left(\text{0.4,0.5}\right);\\\:0.7\end{array}\right)$$
$\:{\varvec{\mathcal {P}}}_{4}$	$$\:\left(\begin{array}{c}\left(\text{0.7,0.2}\right);\\\:0.7\end{array}\right)$$	$$\:\left(\begin{array}{c}\left(\text{0.6,0.3}\right);\\\:0.3\end{array}\right)$$	$$\:\left(\begin{array}{c}\left(\text{0.1,0.7}\right);\\\:0.6\end{array}\right)$$	$$\:\left(\begin{array}{c}\left(\text{0.2,0.4}\right);\\\:0.6\end{array}\right)$$
$\:{\varvec{\mathcal {P}}}_{5}$	$$\:\left(\begin{array}{c}\left(\text{0.4,0.3}\right);\\\:0.8\end{array}\right)$$	$$\:\left(\begin{array}{c}\left(\text{0.3,0.4}\right);\\\:0.7\end{array}\right)$$	$$\:\left(\begin{array}{c}\left(\text{0.2,0.5}\right);\\\:0.3\end{array}\right)$$	$$\:\left(\begin{array}{c}\left(\text{0.3,0.5}\right);\\\:0.9\end{array}\right)$$

**Table 2:** MULTIMOORA results according to dominance theory (PyFNs).

	Ratio system	Reference point	Multiplicative form	MULTIMOORA
$\:{\varvec{\mathcal {P}}}_{1}$	$\:1$	$\:2$	$\:1$	$\:1$
$\:{\varvec{\mathcal {P}}}_{2}$	$\:4$	$\:3$	$\:5$	$\:5$
$\:{\varvec{\mathcal {P}}}_{3}$	$\:3$	$\:5$	$\:3$	$\:3$
$\:{\varvec{\mathcal {P}}}_{4}$	$\:2$	$\:4$	$\:2$	$\:2$
$\:{\varvec{\mathcal {P}}}_{5}$	$\:5$	$\:1$	$\:4$	$\:4$

**Table 3:** Reference point rankings for MULTIMOORA-Cq-ROF PyFNs.

	q-parameters utility score	Reference point
q = 2	$\:{\mathcal {P}}_{1}=0.9201$21, $\:{\mathcal {P}}_{2}=1,{\mathcal {P}}_{3}=1.000358$ $\:{\mathcal {P}}_{4}=0.941508,\:\:{\mathcal {P}}_{5}=0.957878$	$$\begin{gathered} {\mathcal{P}}_{2} > {\mathcal{P}}_{3} > {\mathcal{P}}_{5} \hfill \\ > {\mathcal{P}}_{4} > {\mathcal{P}}_{1} \hfill \\ \end{gathered}$$.
q = 3	$\:{\mathcal {P}}_{1}=0.8814,\:{\mathcal {P}}_{2}=1,\:{\mathcal {P}}_{3}=0.953043$ $\:{\mathcal {P}}_{4}=0.923317,\:\:{\mathcal {P}}_{5}=0.949967$	$$\begin{gathered} {\mathcal{P}}_{2} > {\mathcal{P}}_{3} > {\mathcal{P}}_{5} \hfill \\ > {\mathcal{P}}_{4} > {\mathcal{P}}_{1} \hfill \\ \end{gathered}$$.
q = 4	$\:{\mathcal {P}}_{1}=0.846458,\:{\mathcal {P}}_{2}=1,\:{\mathcal {P}}_{3}=0.916778,\:{\mathcal {P}}_{4}=0.901518,\:{\mathcal {P}}_{5}=0.948182$	$$\begin{gathered} {\mathcal{P}}_{2} > {\mathcal{P}}_{5} > {\mathcal{P}}_{3} \hfill \\ > {\mathcal{P}}_{4} > {\mathcal{P}}_{1} \hfill \\ \end{gathered}$$.
q = 5	$\:{\mathcal {P}}_{1}=0.813146$, $\:{\mathcal {P}}_{2}=$1, $\:{\mathcal {P}}_{3}=0.883919$, $\:{\mathcal {P}}_{4}=0.878574$, $\:{\mathcal {P}}_{5}=0.943268$	$$\begin{gathered} {\mathcal{P}}_{2} > {\mathcal{P}}_{5} > {\mathcal{P}}_{3} \hfill \\ > {\mathcal{P}}_{4} > {\mathcal{P}}_{1} \hfill \\ \end{gathered}$$.

The crisp information is normalized as follows:

$$\:{\stackrel{-}{\rho\:}}_{i}^{RS}=\frac{{\rho\:}_{i}^{RS}}{{ma\mathcalligra {x}}_{i}{\rho\:}_{i}^{RS}}=\left(1,\:\text{0.8823,0.9136},\:0.8378,\:0.9335\right)\:$$

$$\:i=\text{1,2},\dots\:,5.$$

**Phase (6)** For MULTIMOORA-Cq-ROFS, the rank result of the multiplicative utility function: $\:{\mathcal {P}}_{1}>{\mathcal {P}}_{5}>{\mathcal {P}}_{3}>{\mathcal {P}}_{2}>{\mathcal {P}}_{4}$ of are utilized the concept of dominance is used to rank as occurring due to the combination of MOORA and multiplicative form Table 2 provides the MUTIMOORA ranking for $\:q=2$ and $\:\mathcal {K}=2$. The first possibility seems to be the most excellent option. From the MULTIMOORA structure, the is $\:{\mathcal {P}}_{1}>{\mathcal {P}}_{5}>{\mathcal {P}}_{3}>{\mathcal {P}}_{2}>{\mathcal {P}}_{4}$.

Such a prioritized approach to weighted aggregation operators, which was implemented to incorporate Dombi, can drastically enhance the accuracy of decisions about player positioning by providing an alternative aggregation structure capable of dynamically adapting to various dimensions of importance given to performances. By varying the parameter of Dombi, decision-makers can model the relative importance of criteria such as speed, stamina, tactical awareness, and technical skills. This allows for more specific, consistent, and flexible assessments, and thus players are placed in an optimal position in relation to the strategic goals of the teams.

## Impact of different parameters $\varvec{K}$ and $\varvec{q}$ on the MULTIMOORA method based on Cq-ROF and CPYF information

For instance, exceptional examples are compared for $\:q=3$ and $\:\mathcal {K}=2$. These particular examples were picked to illustrate doubts better and to compare with alternative approaches, uncertainties can only be expressed in an intuitionistic if $\:q=1$. The data is more comprehensive, though. The Pythagorean fuzzy set expresses the uncertainty if $\:q=2$. Furthermore, PyFNs already make up the data. However,$\:\:q=3$ was used as an example since Cq-ROF studies examined MADM difficulties from a broader perspective, it is also known that the score values get closer to one another as the $\:q$ values increase.

Let’s analyze how $\:k$ and $\:q$ parameters affect the suggested techniques. Table 4 presents the rankings derived from the parameter effects results on MULTIMOORA-Cq-ROF. There is no aggregating mechanism utilized in the reference point method. It just varies based on $\:q$ values. Consequently, Table 3 provides a reference point method based on $\:q$ values. The impact of the $\:q$ and $\:k$ parameters on the MULTIMOORA-Cq-ROF structure are displayed in Tables 3 and 4. Table 3 illustrates that the optimal choice remains constant based on the $\:q$ values. As the q values rise, the score values get closer to one another. Consequently, the highest limit of $\:q$ is $\:5$. The best player at the position of attacking middle field, according to the MULTIMOORA result, is $\:{\mathcal {P}}_{1}$, which was obtained by applying prioritized aggregation with Dombi TN. For $\:q\:=\:\text{3,4},5$ and $\:\mathcal {K}\:=\:\{\text{1,2},\text{3,5},\text{7,10,15}\}$ values, regardless of the aggregating operators employed. Based on the observed data, the ranking shows that $\:{\mathcal {P}}_{1}$ is the best player in the attacking midfield position. At $\:q=2$, the positions of players $\:{\mathcal {P}}_{1}$ and $\:{\mathcal {P}}_{4}$ are quite close to each other at $\:\mathcal {K}=7,\:10,$ and $\:15$. Still, this closeness does not impact the overall results of the MULTIMOORA method, which continues to rank the player $\:{\mathcal {P}}_{1}$ As the most preferred choice, the method demonstrates its consistency and robustness in evaluating player performance under varying parameter scenarios.

**Table 4:** Impact of different parameters on the MULTIMOORA method.

q-parameter	k values	Ratio system	Multiplicative form	MULTIMOORA method
	$\:\:\:\mathcal {K}=1$	$\:{\mathcal {P}}_{1}>{\mathcal {P}}_{5}>{\mathcal {P}}_{2}>{\mathcal {P}}_{3}>{\mathcal {P}}_{4}$	$\:{\mathcal {P}}_{1}>{\mathcal {P}}_{5}>{\mathcal {P}}_{2}>{\mathcal {P}}_{4}>{\mathcal {P}}_{3}$	$\:{\mathcal {P}}_{1}>{\mathcal {P}}_{5}>{\mathcal {P}}_{2}>{\mathcal {P}}_{3}>{\mathcal {P}}_{4}$
	$\:\mathcal {K}=2$	$\:{\mathcal {P}}_{1}>{\mathcal {P}}_{4}>{\mathcal {P}}_{3}>{\mathcal {P}}_{2}>{\mathcal {P}}_{5}$	$\:{\mathcal {P}}_{1}>{\mathcal {P}}_{4}>{\mathcal {P}}_{5}>{\mathcal {P}}_{2}>{\mathcal {P}}_{3}$	$\:{\mathcal {P}}_{1}>{\mathcal {P}}_{4}>{\mathcal {P}}_{3}>{\mathcal {P}}_{2}>{\mathcal {P}}_{5}$
	$\:\mathcal {K}=3$	$\:{\mathcal {P}}_{1}>{\mathcal {P}}_{4}>{\mathcal {P}}_{3}>{\mathcal {P}}_{2}>{\mathcal {P}}_{5}$	$\:{\mathcal {P}}_{1}>{\mathcal {P}}_{4}>{\mathcal {P}}_{2}>{\mathcal {P}}_{3}>{\mathcal {P}}_{5}$	$\:{\mathcal {P}}_{1}>{\mathcal {P}}_{4}>{\mathcal {P}}_{2}>{\mathcal {P}}_{3}>{\mathcal {P}}_{5}$
	$\:\mathcal {K}=5$	$\:{\mathcal {P}}_{1}>{\mathcal {P}}_{4}>{\mathcal {P}}_{3}>{\mathcal {P}}_{2}>{\mathcal {P}}_{5}$	$\:{\mathcal {P}}_{1}>{\mathcal {P}}_{4}>{\mathcal {P}}_{3}>{\mathcal {P}}_{2}>{\mathcal {P}}_{5}$	$\:{\mathcal {P}}_{1}>{\mathcal {P}}_{4}>{\mathcal {P}}_{3}>{\mathcal {P}}_{2}>{\mathcal {P}}_{5}$
	$\:\mathcal {K}=7$	$\:{\mathcal {P}}_{1}>{\mathcal {P}}_{4}>{\mathcal {P}}_{3}>{\mathcal {P}}_{2}>{\mathcal {P}}_{5}$	$\:{\mathcal {P}}_{1}>{\mathcal {P}}_{4}>{\mathcal {P}}_{3}>{\mathcal {P}}_{2}>{\mathcal {P}}_{5}$	$\:{\mathcal {P}}_{1}>{\mathcal {P}}_{4}>{\mathcal {P}}_{3}>{\mathcal {P}}_{2}>{\mathcal {P}}_{5}$
	$\:\mathcal {K}=10$	$\:{\mathcal {P}}_{1}>{\mathcal {P}}_{4}>{\mathcal {P}}_{3}>{\mathcal {P}}_{2}>{\mathcal {P}}_{5}$	$\:{\mathcal {P}}_{1}>{\mathcal {P}}_{4}>{\mathcal {P}}_{3}>{\mathcal {P}}_{2}>{\mathcal {P}}_{5}$	$\:{\mathcal {P}}_{1}>{\mathcal {P}}_{4}>{\mathcal {P}}_{3}>{\mathcal {P}}_{2}>{\mathcal {P}}_{5}$
	$\:\mathcal {K}=15$	$\:{\mathcal {P}}_{1}>{\mathcal {P}}_{4}>{\mathcal {P}}_{3}>{\mathcal {P}}_{2}>{\mathcal {P}}_{5}$	$\:{\mathcal {P}}_{1}>{\mathcal {P}}_{4}>{\mathcal {P}}_{3}>{\mathcal {P}}_{2}>{\mathcal {P}}_{5}$	$\:{\mathcal {P}}_{1}>{\mathcal {P}}_{4}>{\mathcal {P}}_{3}>{\mathcal {P}}_{2}>{\mathcal {P}}_{5}$
q = 3	$\:\:\:\:\:\mathcal {K}=1$	$\:{\mathcal {P}}_{1}>{\mathcal {P}}_{4}>{\mathcal {P}}_{3}>{\mathcal {P}}_{2}>{\mathcal {P}}_{5}$	$\:{\mathcal {P}}_{1}>{\mathcal {P}}_{5}>{\mathcal {P}}_{2}>{\mathcal {P}}_{4}>{\mathcal {P}}_{3}$	$\:{\mathcal {P}}_{1}>{\mathcal {P}}_{4}>{\mathcal {P}}_{2}>{\mathcal {P}}_{3}>{\mathcal {P}}_{5}$
	$\:\mathcal {K}=2$	$\:{\mathcal {P}}_{1}>{\mathcal {P}}_{4}>{\mathcal {P}}_{3}>{\mathcal {P}}_{2}>{\mathcal {P}}_{5}$	$\:{\mathcal {P}}_{1}>{\mathcal {P}}_{4}>{\mathcal {P}}_{2}>{\mathcal {P}}_{3}>{\mathcal {P}}_{5}$	$\:{\mathcal {P}}_{1}>{\mathcal {P}}_{4}>{\mathcal {P}}_{2}>{\mathcal {P}}_{3}>{\mathcal {P}}_{5}$
	$\:\mathcal {K}=3$	$\:{\mathcal {P}}_{1}>{\mathcal {P}}_{4}>{\mathcal {P}}_{3}>{\mathcal {P}}_{2}>{\mathcal {P}}_{5}$	$\:{\mathcal {P}}_{1}>{\mathcal {P}}_{4}>{\mathcal {P}}_{2}>{\mathcal {P}}_{3}>{\mathcal {P}}_{5}$	$\:{\mathcal {P}}_{1}>{\mathcal {P}}_{4}>{\mathcal {P}}_{2}>{\mathcal {P}}_{3}>{\mathcal {P}}_{5}$
	$\:\mathcal {K}=5$	$\:{\mathcal {P}}_{1}>{\mathcal {P}}_{4}>{\mathcal {P}}_{3}>{\mathcal {P}}_{2}>{\mathcal {P}}_{5}$	$\:{\mathcal {P}}_{1}>{\mathcal {P}}_{4}>{\mathcal {P}}_{2}>{\mathcal {P}}_{3}>{\mathcal {P}}_{5}$	$\:{\mathcal {P}}_{1}>{\mathcal {P}}_{4}>{\mathcal {P}}_{2}>{\mathcal {P}}_{3}>{\mathcal {P}}_{5}$
	$\:\mathcal {K}=7$	$\:{\mathcal {P}}_{1}>{\mathcal {P}}_{4}>{\mathcal {P}}_{3}>{\mathcal {P}}_{2}>{\mathcal {P}}_{5}$	$\:{\mathcal {P}}_{1}>{\mathcal {P}}_{4}>{\mathcal {P}}_{3}>{\mathcal {P}}_{2}>{\mathcal {P}}_{5}$	$\:{\mathcal {P}}_{1}>{\mathcal {P}}_{4}>{\mathcal {P}}_{3}>{\mathcal {P}}_{2}>{\mathcal {P}}_{5}$
	$\:\mathcal {K}=10$	$\:{\mathcal {P}}_{1}>{\mathcal {P}}_{4}>{\mathcal {P}}_{3}>{\mathcal {P}}_{2}>{\mathcal {P}}_{5}$	$\:{\mathcal {P}}_{1}>{\mathcal {P}}_{4}>{\mathcal {P}}_{3}>{\mathcal {P}}_{2}>{\mathcal {P}}_{5}$	$\:{\mathcal {P}}_{1}>{\mathcal {P}}_{4}>{\mathcal {P}}_{3}>{\mathcal {P}}_{2}>{\mathcal {P}}_{5}$
	$\:\mathcal {K}=15$	$\:{\mathcal {P}}_{1}>{\mathcal {P}}_{4}>{\mathcal {P}}_{3}>{\mathcal {P}}_{2}>{\mathcal {P}}_{5}$	$\:{\mathcal {P}}_{1}>{\mathcal {P}}_{5}>{\mathcal {P}}_{3}>{\mathcal {P}}_{2}>{\mathcal {P}}_{5}$	$\:{\mathcal {P}}_{1}>{\mathcal {P}}_{4}>{\mathcal {P}}_{3}>{\mathcal {P}}_{2}>{\mathcal {P}}_{5}$
q = 4	$\:\mathcal {K}=1$	$\:{\mathcal {P}}_{1}>{\mathcal {P}}_{4}>{\mathcal {P}}_{3}>{\mathcal {P}}_{2}>{\mathcal {P}}_{5}$	$\:{\mathcal {P}}_{1}>{\mathcal {P}}_{2}>{\mathcal {P}}_{5}>{\mathcal {P}}_{4}>{\mathcal {P}}_{3}$	$\:{\mathcal {P}}_{1}>{\mathcal {P}}_{4}>{\mathcal {P}}_{2}>{\mathcal {P}}_{3}>{\mathcal {P}}_{5}$
	$\:\mathcal {K}=2$	$\:{\mathcal {P}}_{1}>{\mathcal {P}}_{4}>{\mathcal {P}}_{3}>{\mathcal {P}}_{2}>{\mathcal {P}}_{5}$	$\:{\mathcal {P}}_{1}>{\mathcal {P}}_{4}>{\mathcal {P}}_{2}>{\mathcal {P}}_{3}>{\mathcal {P}}_{5}$	$\:{\mathcal {P}}_{1}>{\mathcal {P}}_{4}>{\mathcal {P}}_{2}>{\mathcal {P}}_{3}>{\mathcal {P}}_{5}$
	$\:\mathcal {K}=3$	$\:{\mathcal {P}}_{1}>{\mathcal {P}}_{4}>{\mathcal {P}}_{3}>{\mathcal {P}}_{2}>{\mathcal {P}}_{5}$	$\:{\mathcal {P}}_{1}>{\mathcal {P}}_{4}>{\mathcal {P}}_{2}>{\mathcal {P}}_{3}>{\mathcal {P}}_{5}$	$\:{\mathcal {P}}_{1}>{\mathcal {P}}_{4}>{\mathcal {P}}_{2}>{\mathcal {P}}_{3}>{\mathcal {P}}_{5}$
	$\:\mathcal {K}=5$	$\:{\mathcal {P}}_{1}>{\mathcal {P}}_{4}>{\mathcal {P}}_{3}>{\mathcal {P}}_{2}>{\mathcal {P}}_{5}$	$\:{\mathcal {P}}_{1}>{\mathcal {P}}_{4}>{\mathcal {P}}_{2}>{\mathcal {P}}_{3}>{\mathcal {P}}_{5}$	$\:{\mathcal {P}}_{1}>{\mathcal {P}}_{4}>{\mathcal {P}}_{2}>{\mathcal {P}}_{3}>{\mathcal {P}}_{5}$
	$\:\mathcal {K}=7$	$\:{\mathcal {P}}_{1}>{\mathcal {P}}_{4}>{\mathcal {P}}_{3}>{\mathcal {P}}_{2}>{\mathcal {P}}_{5}$	$\:{\mathcal {P}}_{1}>{\mathcal {P}}_{4}>{\mathcal {P}}_{3}>{\mathcal {P}}_{2}>{\mathcal {P}}_{5}$	$\:{\mathcal {P}}_{1}>{\mathcal {P}}_{4}>{\mathcal {P}}_{2}>{\mathcal {P}}_{3}>{\mathcal {P}}_{5}$
	$\:\mathcal {K}=10$	$\:{\mathcal {P}}_{1}>{\mathcal {P}}_{4}>{\mathcal {P}}_{3}>{\mathcal {P}}_{2}>{\mathcal {P}}_{5}$	$\:{\mathcal {P}}_{1}>{\mathcal {P}}_{4}>{\mathcal {P}}_{3}>{\mathcal {P}}_{2}>{\mathcal {P}}_{5}$	$\:{\mathcal {P}}_{1}>{\mathcal {P}}_{4}>{\mathcal {P}}_{3}>{\mathcal {P}}_{2}>{\mathcal {P}}_{5}$
	$\:\mathcal {K}=15$	$\:{\mathcal {P}}_{1}>{\mathcal {P}}_{4}>{\mathcal {P}}_{3}>{\mathcal {P}}_{2}>{\mathcal {P}}_{5}$	$\:{\mathcal {P}}_{1}>{\mathcal {P}}_{4}>{\mathcal {P}}_{3}>{\mathcal {P}}_{4}>{\mathcal {P}}_{5}$	$\:{\mathcal {P}}_{1}>{\mathcal {P}}_{4}>{\mathcal {P}}_{3}>{\mathcal {P}}_{2}>{\mathcal {P}}_{5}$
q = 5	$\:\mathcal {K}=1$	$\:{\mathcal {P}}_{1}>{\mathcal {P}}_{4}>{\mathcal {P}}_{3}>{\mathcal {P}}_{2}>{\mathcal {P}}_{5}$	$\:{\mathcal {P}}_{1}>{\mathcal {P}}_{2}>{\mathcal {P}}_{4}>{\mathcal {P}}_{5}>{\mathcal {P}}_{3}$	$\:{\mathcal {P}}_{1}>{\mathcal {P}}_{4}>{\mathcal {P}}_{2}>{\mathcal {P}}_{3}>{\mathcal {P}}_{5}$
	$\:\mathcal {K}=2$	$\:{\mathcal {P}}_{1}>{\mathcal {P}}_{4}>{\mathcal {P}}_{3}>{\mathcal {P}}_{2}>{\mathcal {P}}_{5}$	$\:{\mathcal {P}}_{1}>{\mathcal {P}}_{4}>{\mathcal {P}}_{2}>{\mathcal {P}}_{3}>{\mathcal {P}}_{5}$	$\:{\mathcal {P}}_{1}>{\mathcal {P}}_{4}>{\mathcal {P}}_{2}>{\mathcal {P}}_{3}>{\mathcal {P}}_{5}$
	$\:\mathcal {K}=3$	$\:{\mathcal {P}}_{1}>{\mathcal {P}}_{4}>{\mathcal {P}}_{3}>{\mathcal {P}}_{2}>{\mathcal {P}}_{5}$	$\:{\mathcal {P}}_{1}>{\mathcal {P}}_{4}>{\mathcal {P}}_{2}>{\mathcal {P}}_{3}>{\mathcal {P}}_{5}$	$\:{\mathcal {P}}_{1}>{\mathcal {P}}_{4}>{\mathcal {P}}_{2}>{\mathcal {P}}_{3}>{\mathcal {P}}_{5}$
	$\:\mathcal {K}=5$	$\:{\mathcal {P}}_{1}>{\mathcal {P}}_{4}>{\mathcal {P}}_{3}>{\mathcal {P}}_{2}>{\mathcal {P}}_{5}$	$\:{\mathcal {P}}_{1}>{\mathcal {P}}_{4}>{\mathcal {P}}_{2}>{\mathcal {P}}_{3}>{\mathcal {P}}_{5}$	$\:{\mathcal {P}}_{1}>{\mathcal {P}}_{4}>{\mathcal {P}}_{2}>{\mathcal {P}}_{3}>{\mathcal {P}}_{5}$
	$\:\mathcal {K}=7$	$\:{\mathcal {P}}_{1}>{\mathcal {P}}_{4}>{\mathcal {P}}_{3}>{\mathcal {P}}_{2}>{\mathcal {P}}_{5}$	$\:{\mathcal {P}}_{1}>{\mathcal {P}}_{4}>{\mathcal {P}}_{2}>{\mathcal {P}}_{3}>{\mathcal {P}}_{5}$	$\:{\mathcal {P}}_{1}>{\mathcal {P}}_{4}>{\mathcal {P}}_{2}>{\mathcal {P}}_{3}>{\mathcal {P}}_{5}$
	$\:\mathcal {K}=10$	$\:{\mathcal {P}}_{1}>{\mathcal {P}}_{4}>{\mathcal {P}}_{3}>{\mathcal {P}}_{2}>{\mathcal {P}}_{5}$	$\:{\mathcal {P}}_{1}>{\mathcal {P}}_{4}>{\mathcal {P}}_{2}>{\mathcal {P}}_{3}>{\mathcal {P}}_{5}$	$\:{\mathcal {P}}_{1}>{\mathcal {P}}_{4}>{\mathcal {P}}_{2}>{\mathcal {P}}_{3}>{\mathcal {P}}_{5}$
	$\:\mathcal {K}=15$	$\:{\mathcal {P}}_{1}>{\mathcal {P}}_{4}>{\mathcal {P}}_{3}>{\mathcal {P}}_{2}>{\mathcal {P}}_{5}$	$\:{\mathcal {P}}_{1}>{\mathcal {P}}_{4}>{\mathcal {P}}_{2}>{\mathcal {P}}_{3}>{\mathcal {P}}_{5}$	$\:{\mathcal {P}}_{1}>{\mathcal {P}}_{4}>{\mathcal {P}}_{2}>{\mathcal {P}}_{3}>{\mathcal {P}}_{5}$

## Comparative analysis

This section explores the impact of excluding the circular radius information in the decision-making process by comparing Cq-ROFDPWA and Cq-ROFDPWG operators with q-ROFDPWA and q-ROFDPWG operators. Initially, the decision matrix is aggregated using the Cq-ROFDPWA and Cq-ROFDPWG operators. Cq-ROFDPWA and Cq-ROFDPWG scores are comparatively steady because of the existence of the circular part. In contrast, deleting the radius component and then re-aggregating the resultant data using q-ROFDPWA and q-ROFDPWG leads to a significant increase in the variability of the scores. Particularly, q-ROFDPWA has greater average values, but q-ROFDPWG shows higher shifts, both positive and negative differences, with Cq-based alternatives increasing notably. This is reflective of the stability that the radius component creates; the neglect of which creates increased variances, particularly in the geometric aggregation. An observation of these aggregate techniques reveals that by removing the radius, the smoothing effect is reduced by the weighted averaging operator, and the sensitivity of the geometric operator is increased, especially in q-ROFDPWG, which has remarkably noticeable fluctuations. The derived rankings confirm the significance of the radius element; the formulations with Cq provide more uniform and consistent results as compared to the q-based formulations, which, due to the absence of radius, enhance the variations. This demonstrates the significant role of the radius term in balancing and stabilizing aggregation results, particularly in player evaluation and movement analysis. The empirical assessment discussed in the manuscript proves that the proposed Cq-based operators not only provide mathematically stable aggregation outcomes but also lead to attractive practical benefits. They serve as consistent and continuous player evaluation, significantly enhancing tactical decision-making and real-time positioning optimization. This enhancement is particularly notable when compared to singular, usually q-ROF-based approaches.

**Fig. 3:**
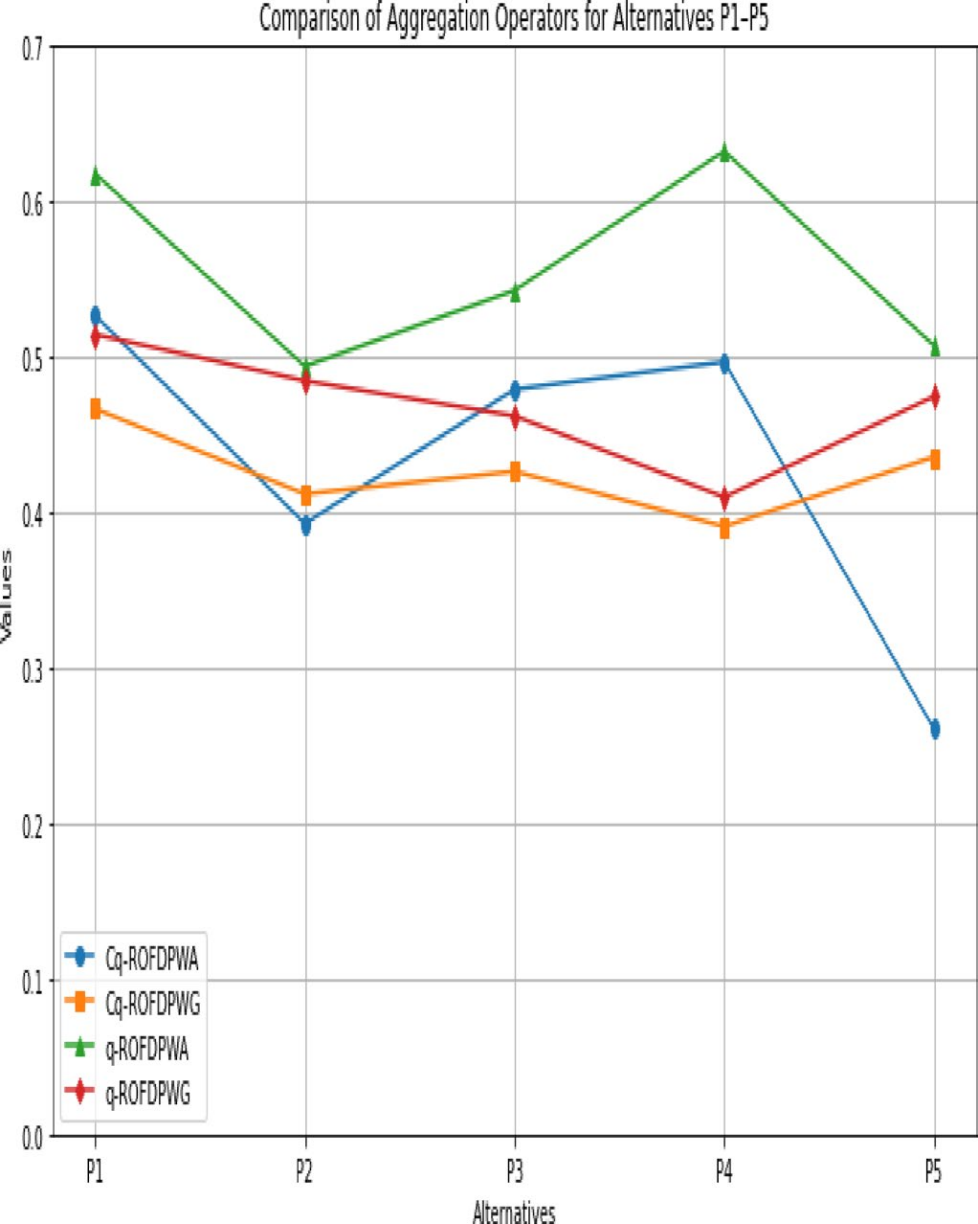
Diagram of Computed Results Using the Cq-ROFDPWA, Cq-ROFDPWG, q-ROFDPWA, and q-ROFDPWG Operators.

We can analyze aggregated results and the ranking of alternatives listed in Tables 5 and 6. Figure 3 displays an analysis of four different aggregation operators: q-ROFDPWA, Cq-ROFDPWG, q-ROFDPWG, and q-ROFDPWS, along with alternatives $\:{\mathcal {P}}_{1}$ to $\:{\mathcal {P}}_{5}$. It is important to note that q-ROFDPWA recorded the highest performance scores on more than one occasion, whereas the results of Cq-ROFDPWG and q-ROFDPWG were in the middle yet quite consistent, thus signifying different operation styles on the part of the operators.

**Table 5:** Score values corresponding to each alternative.

q-ROFDPWA	q-ROFDPWG
0.6182	0.5146
0.4941	0.4848
0.5430	0.4622
0.6326	0.4099
0.5074	0.4748

**Table 6:** Ranking of alternatives.

	Ranked Results
q-ROFDPWA	$\:{\varvec{\mathcal {P}}}_{4}>{\varvec{\mathcal {P}}}_{1}>{\varvec{\mathcal {P}}}_{3}>{\varvec{\mathcal {P}}}_{5}>{\varvec{\mathcal {P}}}_{2}$
q-ROFDPWG	$\:{\mathcal {P}}_{1}>{\mathcal {P}}_{2}>{\mathcal {P}}_{5}>{\mathcal {P}}_{3}>{\mathcal {P}}_{4}$

## Conclusion

This article presents an innovative approach to the system of Cq-ROF information and some exceptional cases. These approaches are utilized to resolve real-life applications and decision algorithms of the MULTIMOORA method based on the Cq-ROF context. The following are the outcomes of the investigation on developing a decision support system for evaluating football players’ positions: The MULTIMOORA approach has been effectively applied in the construction of the Decision Support System for Positioning Players in a Football Team. This technique can identify a player’s optimal position in addition to other positions. The coach can get help from the Decision Support System for determining the Position of Football Players utilizing the MULTIMOORA approach in deciding a player’s position based on their abilities. The findings of the manual calculation and the MULTIMOORA method’s computation for this player’s position in the application are identical.

The MULTIMOORA framework, equipped with Dombi prioritized weighted aggregation operators, and the Cq-ROFS constraints in player positioning optimization on a soccer field, represents a notable methodological contribution. However, some limitations are to be discussed. The first is that the procedure is computational since even in its present form, the need to compute the circular radii, as well as the parameters which can be varied in the operators defined by Dombi, adds a significant degree of complexity to the processing act, which is a considerable difficulty considering that it will be necessary to estimate many players at once. Furthermore, although an understandable intention to develop the model with the general set-up of player placement is evident, its direct, reality-based application in other sports and situational position responsibilities may require methodological adjustments to accommodate the contingency of sports-based features, tactical privileges, and, accordingly, performance levels. Future studies must therefore aim to refine the calculations involved and generally test the effectiveness of the technique in a wide range of sporting settings, which will lead to a more practical application of the method.

In the future, we will expand our developed research work into various fuzzy environments and real-life applications of artificial intelligence, machine learning, social and environmental sciences, medical diagnosis, pattern recognition, and waste management. We can also apply our derived theories to various optimization techniques, such as the TOPSIS method, MARCOS method, EDAS method, and AHP method.

## Supplementary Information

Below is the link to the electronic supplementary material.

Supplementary Material 1

## Data Availability

The datasets used and/or analyzed during the current study are available from the corresponding author upon reasonable request.

## Declarations

### AI declaration statement

The soccer field diagram in this article was generated using an artificial intelligence tool (OpenAI, 2024) under the direct guidance of the authors. No copyrighted or third-party images were used. The figure is fully original and created solely for this scientific publication.Acknowledgement: This work was supported in part by the National Science and Technology Council, Taiwan, under Grant NSTC 114-2410-H-224-001 and internal number 114-1011.
