# Supplementary material for: Integration of MULTIMOORA algorithm combined with circular q-rung orthopair fuzzy information for optimizing player positioning
Source: Sci Rep. 2025 Oct 16;15:36162. doi: 10.1038/s41598-025-18795-0 (PMC12533251; doi:10.1038/s41598-025-18795-0)
Supplement: Supplementary file 1 — Supplementary Material 1 [file 41598_2025_18795_MOESM1_ESM.docx]

Appendix

Appendix A (Proof of Theorem 1)

For$t=2$, we follow the induction method.

$$=\left( \begin{aligned} \left( \begin{aligned} \sqrt[q]{1-\frac{1}{1+\left\{ \begin{aligned} \left( \frac{X_{1}}{\sum_{j=1}^{t} X_{1}} \right)\left( \frac{\mu_{1}^{q}}{1-\mu_{1}^{q}} \right)^{\mathcal{K}} \\ +\left( \frac{X_{2}}{\sum_{j=1}^{t} X_{2}} \right)\left( \frac{\mu_{2}^{q}}{1-\mu_{2}^{q}} \right)^{\mathcal{K}} \end{aligned} \right\}^{1/\mathcal{K}}}}, \\ \sqrt[q]{\frac{1}{1+\left\{ \begin{aligned} \left( \frac{X_{1}}{\sum_{j=1}^{t} X_{1}} \right)\left( \frac{\upsilon_{1}^{q}}{1-\mu_{1}^{q}} \right)^{\mathcal{K}} \\ +\left( \frac{X_{2}}{\sum_{j=1}^{t} X_{2}} \right)\left( \frac{1-\upsilon_{2}^{q}}{\upsilon_{2}^{q}} \right)^{\mathcal{K}} \end{aligned} \right\}^{1/\mathcal{K}}}} \end{aligned} \right); \\ \sqrt[q]{1-\frac{1}{1+\left\{ \begin{aligned} \left( \frac{X_{1}}{\sum_{j=1}^{t} X_{1}} \right)\left( \frac{\mathcal{r}_{1}^{q}}{1-\mathcal{r}_{1}^{q}} \right)^{\mathcal{K}} \\ +\left( \frac{X_{2}}{\sum_{j=1}^{t} X_{2}} \right)\left( \frac{\mathcal{r}_{2}^{q}}{1-\mathcal{r}_{2}^{q}} \right)^{\mathcal{K}} \end{aligned} \right\}^{1/\mathcal{K}}}} \end{aligned} \right)$$

$$\begin{aligned} =\left( \begin{aligned} \left( \begin{aligned} \sqrt[q]{1-\frac{1}{1+\left\{ \sum_{j=1}^{2} \left( \frac{X_{j}}{\sum_{j=1}^{t} X_{j}} \right)\left( \frac{\mu_{j}^{q}}{1-\mu_{j}^{q}} \right)^{\mathcal{K}} \right\}^{\frac{1}{\mathcal{K}}}}}, \\ \sqrt[q]{\frac{1}{1+\left\{ \sum_{j=1}^{2} \left( \frac{X_{j}}{\sum_{j=1}^{t} X_{j}} \right)\left( \frac{1-\upsilon_{j}^{q}}{\upsilon_{j}^{q}} \right)^{\mathcal{K}} \right\}^{\frac{1}{\mathcal{K}}}}} \end{aligned} \right); \\ \sqrt[q]{1-\frac{1}{1+\left\{ \sum_{j=1}^{t} \left( \frac{X_{j}}{\sum_{j=1}^{t} X_{j}} \right)\left( \frac{\mathcal{r}_{j}^{q}}{1-\mathcal{r}_{j}^{q}} \right)^{\mathcal{K}} \right\}^{\frac{1}{\mathcal{K}}}}} \end{aligned} \right) \#\left( 8 \right) \end{aligned}$$

So, Eq. 7 is true for $t=2.$

Let Eq. 7 is true for $t=p$. Then we have:

$$Cq-ROFDPA\left( P_{1},P_{2},\ldots, P_{t} \right)=\oplus_{j=1}^{p}\left( \frac{X_{j}}{\sum_{j=1}^{t} X_{j}}P_{j} \right)$$

$$\begin{aligned} =\left( \begin{aligned} \left( \begin{aligned} \sqrt[q]{1-\frac{1}{1+\left\{ \sum_{j=1}^{p} \left( \frac{X_{j}}{\sum_{j=1}^{t} X_{j}} \right)\left( \frac{\mu_{j}^{q}}{1-\mu_{j}^{q}} \right)^{\mathcal{K}} \right\}^{\frac{1}{\mathcal{K}}}}}, \\ \sqrt[q]{\frac{1}{1+\left\{ \sum_{j=1}^{p} \left( \frac{X_{j}}{\sum_{j=1}^{t} X_{j}} \right)\left( \frac{1-\upsilon_{j}^{q}}{\upsilon_{j}^{q}} \right)^{\mathcal{K}} \right\}^{\frac{1}{\mathcal{K}}}}} \end{aligned} \right); \\ \sqrt[q]{1-\frac{1}{1+\left\{ \sum_{j=1}^{p} \left( \frac{X_{j}}{\sum_{j=1}^{t} X_{j}} \right)\left( \frac{\mathcal{r}_{j}^{q}}{1-\mathcal{r}_{j}^{q}} \right)^{\mathcal{K}} \right\}^{\frac{1}{\mathcal{K}}}}} \end{aligned} \right) \#\left( 9 \right) \end{aligned}$$

Now, we must show it is true for$t=p+1$, then we have:

$$Cq-ROFDPA\left( P_{1},P_{2},\ldots, P_{\mathcal{K}},P_{\mathcal{K}+1} \right)=\oplus_{j=1}^{p}\left( \frac{X_{j}}{\sum_{j=1}^{t} X_{j}}P_{j} \right)\bigoplus\left( \frac{X_{j}}{\sum_{j=1}^{t} X_{j}}P_{p+1} \right)$$

$$=\left( \begin{aligned} \left( \begin{aligned} \sqrt[q]{1-\frac{1}{1+\left\{ \sum_{j=1}^{p} \left( \frac{X_{j}}{\sum_{j=1}^{t} X_{j}} \right)\left( \frac{\mu_{j}^{q}}{1-\mu_{j}^{q}} \right)^{\mathcal{K}} \right\}^{\frac{1}{\mathcal{K}}}}}, \\ \sqrt[q]{\frac{1}{1+\left\{ \sum_{j=1}^{p} \left( \frac{X_{j}}{\sum_{j=1}^{t} X_{j}} \right)\left( \frac{1-\upsilon_{j}^{q}}{\upsilon_{j}^{q}} \right)^{\mathcal{K}} \right\}^{\frac{1}{\mathcal{K}}}}} \end{aligned} \right); \\ \sqrt[q]{1-\frac{1}{1+\left\{ \sum_{j=1}^{p} \left( \frac{X_{j}}{\sum_{j=1}^{t} X_{j}} \right)\left( \frac{\mathcal{r}_{j}^{q}}{1-\mathcal{r}_{j}^{q}} \right)^{\mathcal{K}} \right\}^{\frac{1}{\mathcal{K}}}}} \end{aligned} \right)$$

$$\bigoplus\left( \begin{aligned} \left( \begin{aligned} \sqrt[q]{1-\frac{1}{1+\left\{ \sum_{j=1}^{p} \left( \frac{X_{p+1}}{\sum_{j=1}^{p+1} X_{j}} \right)\left( \frac{\mu_{p+1}^{q}}{1-\mu_{p+1}^{q}} \right)^{\mathcal{K}} \right\}^{1/\mathcal{K}}}}, \\ \sqrt[q]{\frac{1}{1+\left\{ \sum_{j=1}^{p} \left( \frac{X_{p+1}}{\sum_{j=1}^{t} X_{j}} \right)\left( \frac{1-\upsilon_{p+1}^{q}}{\upsilon_{p+1}^{q}} \right)^{\mathcal{K}} \right\}^{1/\mathcal{K}}}} \end{aligned} \right); \\ \sqrt[q]{1-\frac{1}{1+\left\{ \sum_{j=1}^{p} \left( \frac{X_{p+1}}{\sum_{j=1}^{p+1} X_{j}} \right)\left( \frac{\mathcal{r}_{p+1}^{q}}{1-\mathcal{r}_{p+1}^{q}} \right)^{\mathcal{K}} \right\}^{1/\mathcal{K}}}} \end{aligned} \right)$$

$$\begin{aligned} =\left( \begin{aligned} \left( \begin{aligned} \sqrt[q]{1-\frac{1}{1+\left\{ \sum_{j=1}^{p+1} \left( \frac{X_{j}}{\sum_{j=1}^{t} X_{j}} \right)\left( \frac{\mu_{j}^{q}}{1-\mu_{j}^{q}} \right)^{\mathcal{K}} \right\}^{\frac{1}{\mathcal{K}}}}}, \\ \sqrt[q]{\frac{1}{1+\left\{ \sum_{j=1}^{p+1} \left( \frac{X_{j}}{\sum_{j=1}^{t} X_{j}} \right)\left( \frac{1-\upsilon_{j}^{q}}{\upsilon_{j}^{q}} \right)^{\mathcal{K}} \right\}^{\frac{1}{\mathcal{K}}}}} \end{aligned} \right); \\ \sqrt[q]{1-\frac{1}{1+\left\{ \sum_{j=1}^{p+1} \left( \frac{X_{j}}{\sum_{j=1}^{t} X_{j}} \right)\left( \frac{\mathcal{r}_{j}^{q}}{1-\mathcal{r}_{j}^{q}} \right)^{\mathcal{K}} \right\}^{\frac{1}{\mathcal{K}}}}} \end{aligned} \right) \#\left( 10 \right) \end{aligned}$$

Finally, we have proved that Eq. 7 also true for $t=p+1.$

Appendix B (Proof of Theorem 2)

We know that $P_{j}=\left( \left( \mu_{j},\upsilon_{j} \right);\mathcal{r}_{j} \right)$=P, for all $j.$ Considering Eq. 8

$$Cq-ROFDPA\left( P_{1},P_{2},\ldots, P_{t} \right)=\oplus_{j=1}^{t}\left( \frac{X_{j}}{\sum_{j=1}^{t} X_{j}}P_{j} \right)$$

$=\left( \begin{aligned} \left( \begin{aligned} \sqrt[q]{1-\frac{1}{1+\left\{ \sum_{j=1}^{t} \left( \frac{X_{j}}{\sum_{j=1}^{t} X_{j}} \right)\left( \frac{\mu_{j}^{q}}{1-\mu_{j}^{q}} \right)^{\mathcal{K}} \right\}^{\frac{1}{\mathcal{K}}}}}, \\ \sqrt[q]{\frac{1}{1+\left\{ \sum_{j=1}^{t} \left( \frac{X_{j}}{\sum_{j=1}^{t} X_{j}} \right)\left( \frac{1-\upsilon_{j}^{q}}{\upsilon_{j}^{q}} \right)^{\mathcal{K}} \right\}^{1/\mathcal{K}}}} \end{aligned} \right); \\ \sqrt[q]{1-\frac{1}{1+\left\{ \sum_{j=1}^{t} \left( \frac{X_{j}}{\sum_{j=1}^{t} X_{j}} \right)\left( \frac{\mathcal{r}_{j}^{q}}{1-\mathcal{r}_{j}^{q}} \right)^{\mathcal{K}} \right\}^{1/\mathcal{K}}}} \end{aligned} \right)$, for all $j$.

$$=\left( \begin{aligned} \left( \begin{aligned} \sqrt[q]{1-\frac{1}{1+\left\{ \left( \frac{\mu_{j}^{q}}{1-\mu_{j}^{q}} \right)^{\mathcal{K}} \right\}^{\frac{1}{\mathcal{K}}}}}, \\ \sqrt[q]{\frac{1}{1+\left\{ \left( \frac{1-\upsilon_{j}^{q}}{\upsilon_{j}^{q}} \right)^{\mathcal{K}} \right\}^{\frac{1}{\mathcal{K}}}}} \end{aligned} \right); \\ \sqrt[q]{1-\frac{1}{1+\left\{ \left( \frac{\mathcal{r}_{j}^{q}}{1-\mathcal{r}_{j}^{q}} \right)^{\mathcal{K}} \right\}^{1/\mathcal{K}}}} \end{aligned} \right)$$

$$=\left( \begin{aligned} \left( \sqrt[q]{1-\frac{1}{1+\frac{\mu_{j}^{q}}{1-\mu_{j}^{q}}}},\sqrt[q]{\frac{1}{1+\frac{1-\upsilon_{j}^{q}}{\upsilon_{j}^{q}}}} \right); \\ \sqrt[q]{1-\frac{1}{1+\left\{ \left( \frac{\mathcal{r}_{j}^{q}}{1-\mathcal{r}_{j}^{q}} \right)^{\mathcal{K}} \right\}^{1/\mathcal{K}}}} \end{aligned} \right)$$

=$\left( \mu,\upsilon\right)=P.$

$$\begin{aligned} Cq-ROFDPA\left( P_{1},P_{2},\ldots, P_{t} \right)=P \#\left( 11 \right) \end{aligned}$$

Thus, equality (11) is provided.

Appendix C (Proof of Theorem 3)

Let $P^{-}=min\left( P_{1},P_{2},\ldots, P_{t} \right)=(\mu^{-},\upsilon^{-})$ and $P^{+}=max\left( P_{1},P_{2},\ldots, P_{t} \right)=\left( \mu^{+},\upsilon^{+} \right),$ where, $\mu^{-}={min}_{j} \left\{ \mu_{j} \right\},\upsilon^{-}={max}_{j}\left\{ \upsilon_{j} \right\},$ and, $\mu^{+}={max}_{j} \left\{ \mu_{j} \right\},\upsilon^{+}={min}_{j}\left\{ \upsilon_{j} \right\}$. To prove the above expression, we must prove the following inequalities as follows:

$$\sqrt[q]{1-\frac{1}{1+\left\{ \sum_{j=1}^{t} \left( \frac{X_{j}}{\sum_{j=1}^{t} X_{j}} \right)\left( \frac{{\mu_{j}^{q}}^{-}}{1-{\mu_{j}^{q}}^{-}} \right)^{\mathcal{K}} \right\}^{1/\mathcal{K}}}}\leq\sqrt[q]{1-\frac{1}{1+\left\{ \sum_{j=1}^{t} \left( \frac{X_{j}}{\sum_{j=1}^{t} X_{j}} \right)\left( \frac{{\mu_{j}^{q}}^{+}}{1-{\mu_{j}^{q}}^{+}} \right)^{\mathcal{K}} \right\}^{1/\mathcal{K}}}}\leq\sqrt[q]{1-\frac{1}{1+\left\{ \sum_{j=1}^{t} \left( \frac{X_{j}}{\sum_{j=1}^{t} X_{j}} \right)\left( \frac{{\mu_{j}^{q}}^{+}}{1-{\mu_{j}^{q}}^{+}} \right)^{\mathcal{K}} \right\}^{1/\mathcal{K}}}}$$

Another hand, For non-membership degrees.

$$\sqrt[q]{\frac{1}{1+\left\{ \sum_{j=1}^{t} \left( \frac{X_{j}}{\sum_{j=1}^{t} X_{j}} \right)\left( \frac{{\upsilon_{j}^{q}}^{-}}{1-{\mu_{j}^{q}}^{-}} \right)^{\mathcal{K}} \right\}^{1/\mathcal{K}}}}\leq\sqrt[q]{\frac{1}{1+\left\{ \sum_{j=1}^{t} \left( \frac{X_{j}}{\sum_{j=1}^{t} X_{j}} \right)\left( \frac{{\upsilon_{j}^{q}}^{-}}{1-{\mu_{j}^{q}}^{-}} \right)^{\mathcal{K}} \right\}^{1/\mathcal{K}}}}\leq\sqrt[q]{\frac{1}{1+\left\{ \sum_{j=1}^{t} \left( \frac{X_{j}}{\sum_{j=1}^{t} X_{j}} \right)\left( \frac{{\upsilon_{j}^{q}}^{+}}{1-{\mu_{j}^{q}}^{+}} \right)^{\mathcal{K}} \right\}^{1/\mathcal{K}}}}$$

$$\begin{aligned} P^{-}\leq Cq-ROFDPA\left( P_{1},P_{2},\ldots, P_{t} \right)\leq P^{+} \#\left( 12 \right) \end{aligned}$$

**Appendix D (Proof of Theorem 4)**

Let $P_{i}$ and $P_{i}^{'}(j=1,2,\ldots,t)$ be two collections of Cq-ROFNs. If $P_{1}^{,},P_{2}^{,},\ldots, P_{t}^{,}$ is the permutation of $P_{1},P_{2},\ldots, P_{t}$ for all $j$. $X_{j}$ $=\prod_{i=1}^{j-1} S\left( P_{i} \right)\left( j=2,3,\ldots,t \right).$ $X_{j}^{'}=\prod_{i=1}^{j-1} S\left( P_{i}^{,} \right)(j=2,3,\ldots,t)$ and $X_{1}=X_{1}^{'}=1.S\left( P_{i} \right)$ and $S\left( P_{i}^{'} \right)$ are values score function $P_{i}$ and $P_{i}^{'}$ respectively. So, if $P_{j}\leq P_{j}^{'}$. The following inequalities are provided as follows:

$$\begin{aligned} \boldsymbol{Cq-ROFDPA}\left( \boldsymbol{P}_{\boldsymbol{1}}\boldsymbol{,}\boldsymbol{P}_{\boldsymbol{2}}\boldsymbol{,\ldots,}\boldsymbol{P}_{\boldsymbol{t}} \right)\boldsymbol{\leq} \\ \boldsymbol{Cq-ROFDPA}\left( \boldsymbol{If}\boldsymbol{P}_{\boldsymbol{1}}^{\boldsymbol{,}}\boldsymbol{,}\boldsymbol{P}_{\boldsymbol{2}}^{\boldsymbol{,}}\boldsymbol{,\ldots,}\boldsymbol{P}_{\boldsymbol{t}}^{\boldsymbol{,}} \right)\boldsymbol{\#}\left( \boldsymbol{13} \right) \end{aligned}$$

Appendix E (Proof of Theorem 5)

If$t=2$, Eq. 15 obtained as follows:

$$\begin{aligned} =\left( \begin{aligned} \left( \begin{aligned} \sqrt[q]{\frac{1}{1+\left\{ \begin{aligned} \left( \frac{X_{1}}{\sum_{j=1}^{t} X_{j}} \right)\left( \frac{1-\mu_{1}^{q}}{\mu_{1}^{q}} \right)^{\mathcal{K}} \\ +\left( \frac{X_{2}}{\sum_{j=1}^{t} X_{j}} \right)\left( \frac{1-\mu_{2}^{q}}{\mu_{2}^{q}} \right)^{\mathcal{K}} \end{aligned} \right\}^{\frac{1}{\mathcal{K}}}}}, \\ \sqrt[q]{1-\frac{1}{1+\left\{ \begin{aligned} \left( \frac{X_{1}}{\sum_{j=1}^{t} X_{1}} \right)\left( \frac{\upsilon_{1}^{q}}{{1-\upsilon}_{1}^{q}} \right) \\ +\left( \frac{X_{2}}{\sum_{j=1}^{t} X_{j}} \right)\left( \frac{\upsilon_{2}^{q}}{{1-\upsilon}_{2}^{q}} \right)^{\mathcal{K}} \end{aligned} \right\}^{\frac{1}{\mathcal{K}}}}} \end{aligned} \right); \\ \sqrt[q]{\frac{1}{1+\left\{ \begin{aligned} \left( \frac{X_{1}}{\sum_{j=1}^{t} X_{1}} \right)\left( \frac{1-\mathcal{r}_{j}^{q}}{\mathcal{r}_{1}^{q}} \right)^{\mathcal{K}} \\ +\left( \frac{X_{2}}{\sum_{j=1}^{t} X_{j}} \right)\left( \frac{1-\mathcal{r}_{2}^{q}}{\mathcal{r}_{2}^{q}} \right)^{\mathcal{K}} \end{aligned} \right\}^{\frac{1}{\mathcal{K}}}}} \end{aligned} \right) \#\left( 16 \right) \end{aligned}$$

Since, Eq. (15) is true for $t=2.$

Let Eq (16) also true for $t=p$.

$$Cq-ROFDPA\left( P_{1},P_{2},\ldots, P_{\mathcal{K}} \right)=\oplus_{j=1}^{P}\left( \frac{X_{j}}{\sum_{j=1}^{t} X_{j}}P_{j} \right)$$

$$\begin{aligned} =\left( \begin{aligned} \left( \begin{aligned} \sqrt[q]{\frac{1}{1+\left\{ \sum_{j=1}^{p} \left( \frac{X_{j}}{\sum_{j=1}^{t} X_{j}} \right)\left( \frac{1-\mu_{j}^{q}}{\mu_{j}^{q}} \right)^{\mathcal{K}} \right\}^{\frac{1}{\mathcal{K}}}}}, \\ \sqrt[q]{1-\frac{1}{1+\left\{ \sum_{j=1}^{p} \left( \frac{X_{j}}{\sum_{j=1}^{t} X_{j}} \right)\left( \frac{\upsilon_{j}^{q}}{{1-\upsilon}_{j}^{q}} \right)^{\mathcal{K}} \right\}^{\frac{1}{\mathcal{K}}}}} \end{aligned} \right); \\ \sqrt[q]{\frac{1}{1+\left\{ \sum_{j=1}^{p} \left( \frac{X_{j}}{\sum_{j=1}^{t} X_{j}} \right)\left( \frac{1-\mathcal{r}_{j}^{q}}{\mathcal{r}_{j}^{q}} \right)^{\mathcal{K}} \right\}^{\frac{1}{\mathcal{K}}}}} \end{aligned} \right) \#\left( 17 \right) \end{aligned}$$

In this instance, we must demonstrate that it holds true for t = p + 1.

$$Cq-ROFDPA\left( P_{1},P_{2},\ldots, P_{\mathcal{K}},P_{\mathcal{K}+1} \right)=\oplus_{j=1}^{P}\left( \frac{X_{j}}{\sum_{j=1}^{t} X_{j}}P_{j} \right)\bigoplus\left( \frac{X_{p+1}}{\sum_{j=1}^{t} X_{j}}P_{p+1} \right)$$

$$\begin{aligned} =\left( \begin{aligned} \left( \begin{aligned} \sqrt[q]{\frac{1}{1+\left\{ \sum_{j=1}^{p} \left( \frac{X_{j}}{\sum_{j=1}^{t} X_{j}} \right)\left( \frac{1-\mu_{j}^{q}}{\mu_{j}^{q}} \right)^{\mathcal{K}} \right\}^{\frac{1}{\mathcal{K}}}}}, \\ \sqrt[q]{1-\frac{1}{1+\left\{ \sum_{j=1}^{p} \left( \frac{X_{j}}{\sum_{j=1}^{t} X_{j}} \right)\left( \frac{\upsilon_{j}^{q}}{{1-\upsilon}_{j}^{q}} \right)^{\mathcal{K}} \right\}^{\frac{1}{\mathcal{K}}}}} \end{aligned} \right); \\ \sqrt[q]{\frac{1}{1+\left\{ \sum_{j=1}^{p} \left( \frac{X_{j}}{\sum_{j=1}^{t} X_{j}} \right)\left( \frac{1-\mathcal{r}_{j}^{q}}{\mathcal{r}_{j}^{q}} \right)^{\mathcal{K}} \right\}^{\frac{1}{\mathcal{K}}}}} \end{aligned} \right) \\ \bigoplus\left( \begin{aligned} \left( \begin{aligned} \sqrt[q]{\frac{1}{1+\left\{ \left( \frac{X_{p+1}}{\sum_{j=1}^{P+1} X_{j}} \right)\left( \frac{1-\mu_{p+1}^{q}}{\mu_{P+1}^{q}} \right)^{\mathcal{K}} \right\}^{\frac{1}{\mathcal{K}}}}}, \\ \sqrt[q]{1-\frac{1}{1+\left\{ \left( \frac{X_{p+1}}{\sum_{j=1}^{p+1} X_{j}} \right)\left( \frac{\upsilon_{p+1}^{q}}{{1-\upsilon}_{P+1}^{q}} \right)^{\mathcal{K}} \right\}^{\frac{1}{\mathcal{K}}}}} \end{aligned} \right); \\ \sqrt[q]{\frac{1}{1+\left\{ \left( \frac{X_{p+1}}{\sum_{j=1}^{p+1} X_{j}} \right)\left( \frac{1-\mathcal{r}_{p+1}^{q}}{\mathcal{r}_{p+1}^{q}} \right)^{\mathcal{K}} \right\}^{\frac{1}{\mathcal{K}}}}} \end{aligned} \right)\#\left( 18 \right) \end{aligned}$$

The above expression is valid for $t=p+1$.
